# Vaccine based on folded RBD‐PreS fusion protein with potential to induce sterilizing immunity to SARS‐CoV‐2 variants

**DOI:** 10.1111/all.15305

**Published:** 2022-04-15

**Authors:** Pia Gattinger, Bernhard Kratzer, Inna Tulaeva, Katarzyna Niespodziana, Anna Ohradanova‐Repic, Laura Gebetsberger, Kristina Borochova, Erika Garner‐Spitzer, Doris Trapin, Gerhard Hofer, Walter Keller, Isabella Baumgartner, Ivan Tancevski, Musa Khaitov, Alexander Karaulov, Hannes Stockinger, Ursula Wiedermann, Winfried F. Pickl, Rudolf Valenta

**Affiliations:** ^1^ Department of Pathophysiology and Allergy Research Division of Immunopathology Center for Pathophysiology, Infectiology and Immunology Medical University of Vienna Vienna Austria; ^2^ Center for Pathophysiology, Infectiology and Immunology Institute of Immunology Medical University of Vienna Vienna Austria; ^3^ Laboratory for Immunopathology Department of Clinical Immunology and Allergology Sechenov First Moscow State Medical University Moscow Russia; ^4^ Karl Landsteiner University of Health Sciences Krems Austria; ^5^ Center for Pathophysiology, Infectiology and Immunology Institute for Hygiene and Applied Immunology Medical University of Vienna Vienna Austria; ^6^ Institute of Specific Prophylaxis and Tropical Medicine Medical University of Vienna Vienna Austria; ^7^ Department of Materials and Environmental Chemistry University of Stockholm Stockholm Sweden; ^8^ Institute of Molecular Biosciences, BioTechMed Graz University of Graz Graz Austria; ^9^ Department of Ophthalmology Medical University Vienna Vienna Austria; ^10^ Department of Internal Medicine II Medical University of Innsbruck Innsbruck Austria; ^11^ NRC Institute of Immunology FMBA of Russia Moscow Russia; ^12^ Pirogov Russian National Research Medical University Moscow Russia

**Keywords:** antibody response, COVID‐19, neutralizing antibodies, SARS‐CoV‐2, sterilizing immunity, vaccine

## Abstract

**Background:**

Severe acute respiratory syndrome coronavirus 2 (SARS‐CoV‐2) is responsible for the ongoing global COVID‐19 pandemic. One possibility to control the pandemic is to induce sterilizing immunity through the induction and maintenance of neutralizing antibodies preventing SARS‐CoV‐2 from entering human cells to replicate in.

**Methods:**

We report the construction and *in vitro* and *in vivo* characterization of a SARS‐CoV‐2 subunit vaccine (PreS‐RBD) based on a structurally folded recombinant fusion protein consisting of two SARS‐CoV‐2 Spike protein receptor‐binding domains (RBD) fused to the N‐ and C‐terminus of hepatitis B virus (HBV) surface antigen PreS to enable the two unrelated proteins serving as immunologic carriers for each other.

**Results:**

PreS‐RBD, but not RBD alone, induced a robust and uniform RBD‐specific IgG response in rabbits. Currently available genetic SARS‐CoV‐2 vaccines induce mainly transient IgG_1_ responses in vaccinated subjects whereas the PreS‐RBD vaccine induced RBD‐specific IgG antibodies consisting of an early IgG_1_ and sustained IgG_4_ antibody response in a SARS‐CoV‐2 naive subject. PreS‐RBD‐specific IgG antibodies were detected in serum and mucosal secretions, reacted with SARS‐CoV‐2 variants, including the omicron variant of concern and the HBV receptor‐binding sites on PreS of currently known HBV genotypes. PreS‐RBD‐specific antibodies of the immunized subject more potently inhibited the interaction of RBD with its human receptor ACE2 and their virus‐neutralizing titers (VNTs) were higher than median VNTs in a random sample of healthy subjects fully immunized with registered SARS‐CoV‐2 vaccines or in COVID‐19 convalescent subjects.

**Conclusion:**

The PreS‐RBD vaccine has the potential to serve as a combination vaccine for inducing sterilizing immunity against SARS‐CoV‐2 and HBV by stopping viral replication through the inhibition of cellular virus entry.

## Graphical abstract

- This study reports the design and characterization of a SARS‐CoV‐2 subunit vaccine (PreS‐RBD).
- PreS‐RBD, but not RBD alone, induces RBD‐specific IgG_1_ and long‐lasting IgG_4_ in serum and mucosal fluids, and cross‐reacts with SARS‐CoV‐2 variants of concern.
- PreS‐RBD‐specific antibodies of the immunized subject more potently inhibit the interaction of RBD with angiotensin converting enzyme 2 (ACE2), and their virus‐neutralizing titers are higher than median titers in a random sample of healthy subjects fully immunized with registered SARS‐CoV‐2 vaccines or in COVID‐19 convalescent subjects.

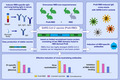

**Abbreviations**
COVID‐19: coronavirus disease 2019
HBV: hepatitis B virus
IgG: immunoglobulin G
PreS: HVB domain
RBD: receptor‐binding domain
SARS‐CoV‐2: severe acute respiratory syndrome coronavirus 2

## 1 INTRODUCTION

The ongoing COVID‐19 pandemic caused by SARS‐CoV‐2 infections continues to be a major challenge to humankind. According to the Johns Hopkins University of Medicine Coronavirus Resource Center, more than 330 million cases and more than 5.5 million COVID‐19‐associated deaths have been reported until January 21, 2022 (https://coronavirus.jhu.edu/map.html). The rapid development of SARS‐CoV‐2‐specific vaccines has reduced the number of weekly reported COVID‐19‐associated deaths by approximately 50% when comparing the numbers from fall/winter 2021 to those observed for the same period in 2020 (https://coronavirus.jhu.edu/map.html). However, despite the fact that nearly 9.7 billion vaccine doses have been administered worldwide by this time, the number of weekly COVID‐19 cases in 2022 has currently doubled compared with the same time in 2021 (https://coronavirus.jhu.edu/map.html).

The currently available COVID‐19 vaccines deviate from established vaccination technologies in that they rely on either the delivery of nucleic acid encoding the SARS‐CoV‐2 Spike protein (S) by adenovirus‐based viral vectors, or by S‐encoding mRNA into human cells, which then produce and release the S antigen triggering an immune response that resembles to some extent that following a SARS‐CoV‐2 infection.^1^ Accordingly, the SARS‐CoV‐2‐specific immune response induced by the aforementioned genetic vaccines is based on MHC class I‐ and MHC class II‐mediated antigen presentation accompanied by S‐specific CD4^+^ and CD8^+^ T‐cell responses and relatively short‐lived S‐specific, mainly IgG_1_ antibody responses^2^, ^3^, ^4^ of which the levels and specificities may vary depending on the amount and quality of S antigen produced by the cells of the vaccinee.^5^ The genetic SARS‐CoV‐2 vaccines could be made available relatively quickly because they by‐pass the need of establishing a process for producing defined protein immunogens and thus represented an important first step toward reducing COVID‐19‐associated deaths. However, immunization with genetic SARS‐CoV‐2 vaccines has also been reported to be associated with adverse events such as thromboembolic events,^6^, ^7^, ^8^ myocarditis,^9^, ^10^, ^11^ anaphylactic reactions,^12^, ^13^ neurological complications,^14^ and deaths considered to be associated with vaccination.^15^ Furthermore, currently available SARS‐CoV‐2 vaccines are challenged by the emergence of novel SARS‐CoV‐2 variants of which certain seem to escape neutralization by vaccine‐induced antibodies.^16^ One new variant B.1.1.529, named omicron, which was first identified in South Africa by the World Health Organization in the middle of November 2021 (WHO COVID‐19 Dashboard https://covid19.who.int/) is of particular concern because it has at least 21 mutations in the part encoding the S1 subunit and 15 amino acid exchanges in the receptor‐binding domain of the S protein, RBD, which is significantly more than previous variants of concern (VOCs).^17^ Indeed, omicron was detected in Asia in mid‐November^18^ and has since become a dominant VOC in all parts of the world.^17^ First reports already indicate that currently available COVID‐19 vaccines may confer less protection against omicron^19^, ^20^, ^21^, ^22^ infection.

Antibodies directed against RBD are important for virus neutralization,^23^, ^24^ correlate with protection in vaccinated subjects^25^ and due to their ability to prevent the virus from entering the human cell and replicating in the host could be a key for obtaining sterilizing immunity.^26^ However, it was found that approximately 20% of COVID‐19 convalescent patients, although producing antibodies to other SARS‐CoV‐2 antigens and epitopes, do not sufficiently produce RBD‐specific IgG antibodies to block the RBD‐ACE2 binding and hence are poor‐ or non‐responders to RBD.^23^, ^24^

Here, we report the construction, expression, and purification, as well as the biochemical and immunological *in vitro* and *in vivo* characterization of a SARS‐CoV‐2 subunit vaccine PreS‐RBD. PreS‐RBD is based on a recombinant fusion protein consisting of the human hepatitis B virus (HBV)‐derived PreS antigen, which by itself or as part of fusion proteins induces human HBV‐protective immune responses^27^, ^28^, ^29^, ^30^ and two SARS‐CoV‐2 RBD domains attached to the N‐ and C‐terminus of PreS. According to the hapten‐carrier principle discovered by Nobel laureate Baruj Benacerraf,^31^ the fusion of RBD to PreS aimed to increase the immunogenicity of RBD. PreS‐RBD was formulated with aluminum hydroxide (alum), an adjuvant which has been safely used both in vaccines against infectious diseases and in therapeutic allergy vaccines (i.e., allergen‐specific immunotherapy, AIT) for decades.^32^ AIT‐induced allergen‐specific IgG responses typically consist of rapidly evolving specific IgG_1_ responses and the late but sustained production of neutralizing allergen‐specific IgG_4_ antibodies, which persist even years after discontinuation of treatment and leads to sustained protection of allergic patients from allergen‐induced allergic inflammation.^33^, ^34^, ^35^ Results obtained for the PreS‐RBD subunit vaccine in this study suggest that PreS‐RBD has several features, which make it an interesting SARS‐CoV‐2 vaccine candidate for inducing sterilizing immunity.

## 2 MATERIALS AND METHODS

Detailed Materials and Methods can be found in the Appendix: Online Repository.

## 3 RESULTS

### 3.1 Characterization of recombinant SARS‐CoV‐2 subunit vaccines based on recombinant PreS‐RBD fusion proteins

**FIGURE 1:**
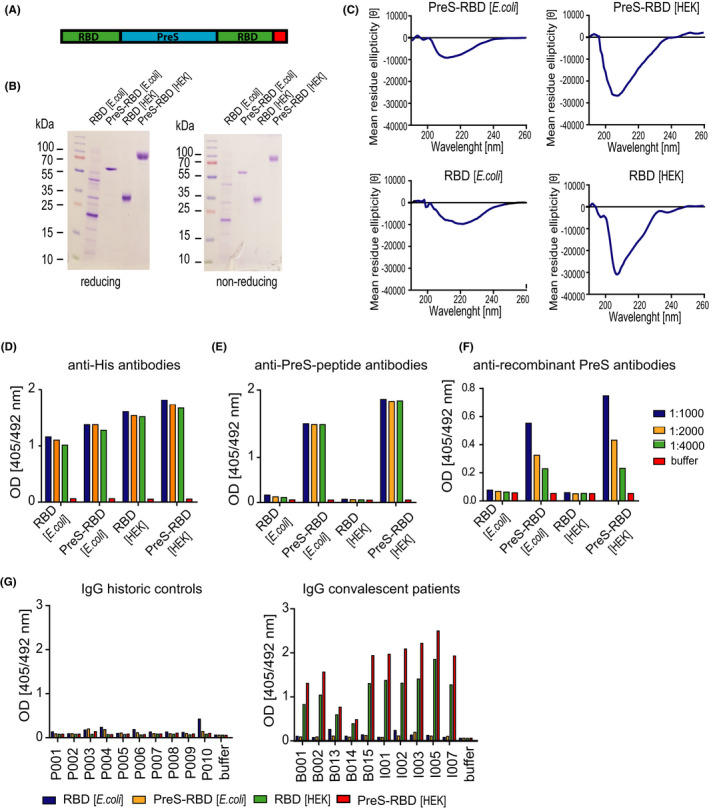
Characterization of the recombinant RBD‐PreS fusion protein. (A) Scheme of the recombinant PreS‐RBD protein with two RBD domains (green) fused to the N‐ and C‐ terminus of PreS (blue), respectively. The C‐terminal hexa‐histidine tag is indicated in red. (B) Coomassie blue‐stained SDS‐PAGE containing *E*. *coli*‐ and HEK cell‐expressed PreS‐RBD and RBD separated under reducing and non‐reducing conditions. Molecular weights are indicated in kDa. (C) Circular dichroism analysis of *E*. *coli*‐ and HEK cell‐expressed PreS‐RBD and RBD. Scans show molecular ellipticities (y‐axes) at given wave lengths (*x*‐axes). Reactivity of *E*. *coli*‐ and HEK cell‐expressed PreS‐RBD and RBD with different dilutions of (D) anti‐His antibodies, (E) anti‐PreS‐peptide antibodies, (F) anti‐recombinant PreS antibodies, and (G) IgG antibodies (1:50 diluted) from COVID‐19‐convalescent subjects (*n* = 10) and historic controls (*n* = 10) by ELISA. OD _(405/492 nm)_ values (*y*‐axes) are average values of duplicate determinations with <5% deviation and correspond to amounts of bound antibodies. Buffer without primary antibodies served as negative control

Our experience in engineering vaccines for allergen‐specific immunotherapy shows that according to the hapten‐carrier principle^31^ one can enhance the ability of a given antigen/peptide to induce specific antibody responses upon immunization by fusing it to an unrelated carrier protein and providing it in more than one copy.^36^, ^37^, ^38^ Accordingly, we designed a fusion protein (PreS‐RBD) consisting of two RBD domains, one fused to the N‐terminus and one fused to the C‐terminus of the human hepatitis B virus (HBV)‐derived PreS, the HBV surface antigen containing the binding site of HBV to the NTCP (sodium taurocholate co‐transporting polypeptide) receptor on hepatocytes^39^, ^40^ (Figure 1A). We have previously found that a vaccine based on PreS‐fusion proteins containing hypoallergenic peptides derived from the major timothy grass pollen allergens (i.e., BM32) induced a robust grass pollen allergen‐specific IgG response protecting grass pollen allergic patients from grass pollen allergy^41^, ^42^ (ClinicalTrials.gov Identifier: NCT02643641). On the contrary, BM32 and one of its components BM325 (i.e., VVX001) induced antibodies in vaccinated subjects preventing HBV infection *in vitro* ^29^, ^30^ and *in vivo*, as recently shown in a therapeutic vaccination study conducted in patients suffering from chronic HBV infection.^43^ Synthetic genes coding for PreS‐RBD and, for control purposes for RBD alone, were codon‐optimized either for the expression in *E*. *coli* or in human cell lines. Expressed proteins were purified via Nickel affinity chromatography via a hexahistidine tag which was added to the recombinant proteins (Figure 1A,B). *E*. *coli*‐expressed PreS‐RBD migrated at approximately 60 kDa in SDS‐PAGE under reducing and non‐reducing conditions whereas the HEK cell‐expressed fusion protein migrated at 70 kDa (Figure 1B). The higher molecular weight of HEK cell‐expressed PreS‐RBD versus *E*. *coli*‐expressed PreS‐RBD is compatible with the presence of six N‐glycosylation sites in the former protein. Likewise, HEK cell‐expressed RBD containing two N‐glycosylation sites had a higher molecular weight (ie 35 kDa) compared to *E*. *coli*‐expressed RBD. *E*. *coli*‐expressed RBD also showed additional bands under reducing and non‐reducing conditions (Figure 1B) which stained with anti‐His antibodies and hence did not represent impurities (data not shown).

The analysis of recombinant RBD proteins regarding the presence of fold and secondary structure by far UV circular dichroism spectroscopy (CD) analyses is presented in Figure 1(C). RBD expressed in HEK cells revealed a minimum at 207 nm, which is consistent with a previous study reporting the expression of functional RBD^44^ resembling a predominant β‐sheet structure. HEK cell‐expressed PreS‐RBD exhibited a minimum at 209 nm which is also indicative of the presence of considerable β‐sheet secondary structure (Figure 1C). *E*. *coli*‐expressed RBD and PreS‐RBD showed a strong reduction of ellipticity and of the corresponding minima indicating the presence of a high proportion of unfolded structure in the proteins (Figure 1C). The unfolded RBD also did not bind to the ACE2 receptor (data not shown).

In a next set of experiments, we characterized recombinant RBD and PreS‐RBD proteins regarding their reactivity with a panel of antibody probes specific for PreS, RBD, and the His‐tag (Figure 1D–G). Figure 1D shows that *E*. *coli*‐ and HEK cell‐expressed RBD and PreS‐RBD reacted with different dilutions of anti‐His antibodies (HEK cell‐expressed PreS‐RBD and RBD > *E. coli*‐expressed PreS‐RBD and RBD). No reaction was observed when the primary anti‐His antibody was omitted (Figure 1D). HEK cell‐ > *E*. *coli*‐expressed PreS‐RBD reacted with antisera raised against PreS‐peptides and against *E*. *coli*‐expressed PreS whereas recombinant RBD proteins did not (Figure 1E,F). No reaction was observed when the primary anti‐PreS antibodies were omitted (Figure 1E,F). Next, we tested sera obtained from subjects before the SARS‐CoV‐2 pandemic (i.e., historic control sera) and sera obtained from COVID‐19 convalescent patients for IgG reactivity with *E*. *coli*‐ and HEK cell‐expressed RBD and PreS‐RBD proteins. Historic control sera showed no IgG reactivity to folded RBD and PreS‐RBD, whereas a few sera (ie P003, P004, and P010) showed low reactivity to unfolded RBD and PreS‐RBD (Figure 1G, left). By contrast, sera from COVID‐19 convalescent patients showed pronounced IgG reactivity to HEK cell‐expressed PreS‐RBD >RBD but no relevant reactivity to *E*. *coli*‐expressed proteins (Figure 1G, right). Only few sera (i.e., B013, I002) showed very weak reactivity to the unfolded bacterially expressed proteins. No reactivity was observed when patients´ sera were omitted (Figure 1G).

### 3.2 Immunization with PreS‐RBD but not with RBD alone induces a uniform RBD‐specific antibody response

**FIGURE 2:**
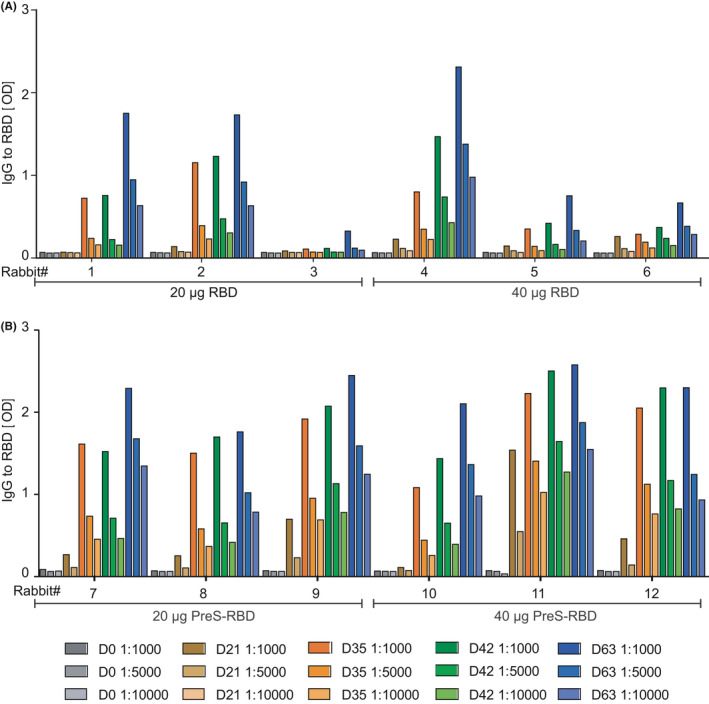
RBD‐specific IgG responses in immunized rabbits. Shown are IgG responses of rabbits, immunized with equimolar RBD doses (20 or 40 µg) of folded RBD monomer or PreS‐RBD. IgG antibody levels specific for folded RBD (y‐axes: OD_405/492nm_ levels) of three rabbits per group, immunized with two equimolar RBD doses (20 or 40 µg) (*x*‐axes) of (A) folded RBD monomer or (B) folded PreS‐RBD are shown for different time points of bleeding and serum dilutions as indicated in the insets. OD_405/492nm_ values are shown as duplicate determinations with <5% deviation

The ability of folded PreS‐RBD or RBD to induce antibody responses was investigated by immunizing rabbits, which allows studying the uniformity of the induced immune responses in out‐bred animals. The choice of out‐bred animals is important because we found that approximately 20% of SARS‐CoV‐2‐infected subjects did not mount RBD‐specific antibodies and hence represented “RBD‐non‐responders”.^24^ Four groups of three rabbits were immunized three times in 3‐week intervals with 20 or 40 µg of alum‐adsorbed RBD or two doses of alum‐adsorbed PreS‐RBD containing equimolar amounts of RBD. Figure 2 shows IgG levels measured by ELISA for three different dilutions of sera of the 12 rabbits. Even after three injections, one (rabbit #3) and two (rabbits #5, #6) out of the six RBD‐immunized animals were non‐ and low‐responders, respectively, whereas each of the six PreS‐RBD‐immunized rabbits developed robust and uniform RBD‐specific IgG levels already after two injections (Figure 2).

### 3.3 Immunization with folded but not with unfolded PreS‐RBD induces antibodies in serum of a COVID‐19 naive subject that cross‐react with SARS‐CoV‐2 variants

**FIGURE 3:**
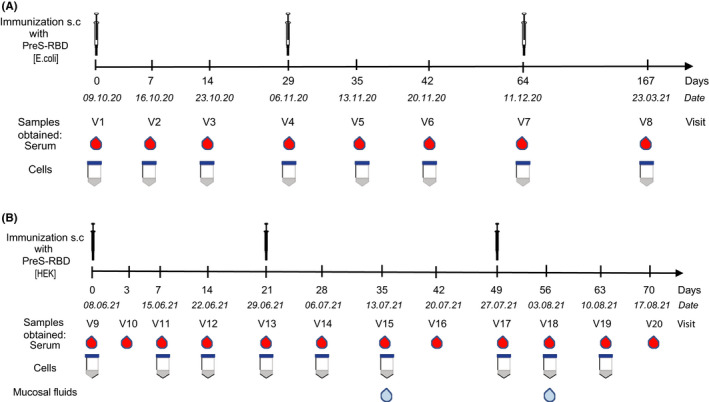
Immunization scheme of a healthy, SARS‐Cov‐2 negative subject indicating time points and dates of injection, sampling (serum, cells, mucosal fluids) during immunization with (A) unfolded *E*. *coli*‐ and (B) folded HEK cell‐expressed PreS‐RBD

**FIGURE 4:**
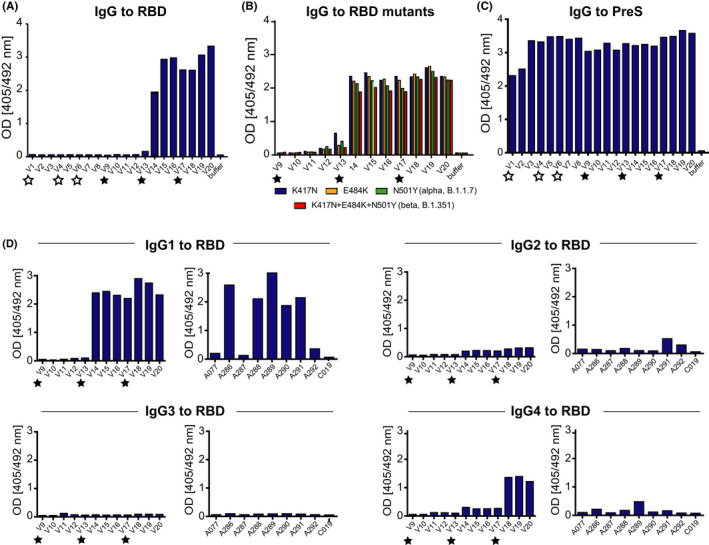
Development of specific antibody responses in the immunized subject. Serum IgG reactivity to (A) folded RBD after immunization with unfolded *E*. *coli* (white stars) and folded HEK cell‐expressed (black stars) PreS‐RBD (x‐axes, time points). (B) IgG reactivity to RBD mutations K417N, E484K, N501Y (alpha, B.1.1.7), and K417N+E484K+N501Y (beta, B.1.351) after immunization with HEK cell‐expressed PreS‐RBD at different time points (*x*‐axis). (C) PreS‐specific IgG after immunization with unfolded *E*. *coli* (white stars) and folded HEK cell‐expressed (black stars) PreS‐RBD (*x*‐axes, time points) and (D) IgG subclass analysis of immunization with HEK cell‐expressed PreS‐RBD (left) at different time points (*x*‐axes) and of subjects (*x*‐axes) 4 weeks after full immunization with licensed COVID‐19 vaccines (right). Sera were diluted 1:50; OD values are average values of duplicate determinations with <5% deviation (*y*‐axes) and correspond to amounts of bound antibodies

Immunization of a SARS‐CoV‐2 naive human subject with unfolded *E*. *coli*‐expressed PreS‐RBD was started on 9 October 2020 by the subject (Figure 3). In total, three subcutaneous injections were administered approximately 4 weeks apart. Figure 4 shows that immunization with unfolded PreS‐RBD did not induce IgG responses against folded HEK cell‐expressed RBD. This result was in agreement with data obtained in rabbits where *E*. *coli*‐expressed PreS‐RBD failed to induce IgG responses against folded RBD.^24^ These results and the finding that only folded RBD induced IgG antibodies against folded RBD in rabbits, which strongly neutralized SARS‐CoV‐2 infections *in vitro* and prevented RBD binding to ACE2^24^ led to the construction of a recombinant PreS‐RBD which was completely identical in its primary sequence as the *E*. *coli*‐expressed version but due to expression in HEK cells was obtained as folded protein (Figure 1). Immunization of the human subject with folded, HEK cell‐expressed PreS‐RBD was initiated on 8 June 2021 by the subject and induced a strong IgG response against folded RBD, as determined 1 week after the second injection (i.e., at visit 14) (Figure 3). Moreover, the RBD‐specific antibodies induced by folded PreS‐RBD based on the Wuhan‐hu‐1 sequence induced IgG antibodies which cross‐reacted equally with SARS‐CoV‐2 variants (Wuhan‐hu‐1, K417N, E484K, alpha, beta, delta, and omicron) (Figure 4A,B, Figures S1–S3A). Of note, also rabbit antibodies induced by immunization with the folded HEK cell‐expressed Wuhan‐hu‐1 PreS‐RBD protein cross‐reacted with SARS‐CoV‐variants delta and omicron (Figure S3B).

Since the volunteer had been previously vaccinated with the PreS‐containing grass pollen allergy vaccine BM325 (i.e., VVX001)^43^ (ClinicalTrials.gov Identifier: NCT03625934), a PreS‐specific IgG response was detected as early as visit 1, which further increased during immunization with *E*. *coli*‐expressed PreS‐RBD and even more so after immunization with folded HEK cell‐expressed PreS‐RBD (Figure 4C). Figure S4 (right upper panel) corroborates this finding by testing different serum dilutions of the subject showing that the PreS‐specific IgG levels indeed further increased after immunization with folded HEK cell‐expressed PreS‐RBD. Figure S4 further shows that the IgG isotype dominated the RBD‐specific antibody responses in the immunized subject and was accompanied by a low IgM response and an IgA response which peaked shortly after the beginning of the immunization. The PreS‐specific antibody response in the subject was dominated by IgG antibodies, some IgM responses but no relevant IgA response (Figure S4).

### 3.4 Immunization with folded PreS‐RBD induces antibodies reacting with the NTCP binding sites of HBV genotypes A‐H

The PreS protein contains at its N‐terminus the binding site of HBV to its receptor NTCP on hepatocytes,^39^ and, therefore, is a candidate vaccine antigen for preventive and therapeutic HBV vaccines.^29^, ^30^, ^40^, ^43^ Figure S5 shows that due to previous vaccination with BM325, a component of BM32, the subject has had IgG antibodies specific for PreS‐derived peptides, in particular to PreS P2 which contains the NTCP binding site of HBV and against peptides including the amino acid sequence crucial for infectivity (PreS aa13–aa51) of HBV genotypes A–H.^30^ Vaccination with three doses of unfolded *E*. *coli*‐expressed PreS‐RBD increased the PreS peptide‐specific IgG responses at visit 9 (Figure 3, Figure S5) as determined approximately half a year after the last vaccination with *E*. *coli*‐expressed PreS‐RBD (i.e., at visit 7). The administration of three doses of folded HEK cell‐expressed PreS‐RBD strongly increased IgG levels to peptides spanning PreS, in particular the N‐terminal peptides containing the NTCP binding site and peptides representing the NTCP binding sites from all 8 HBV genotypes as measured at visit 20, approximately 4 weeks after the third injection (Figure 3, Figure S5).

### 3.5 Immunization with folded PreS‐RBD induces an early RBD‐specific IgG_1_ response followed by a late but sustained IgG_4_ response

We found that immunization with the PreS‐containing grass pollen allergy vaccine BM32 induces a biphasic allergen‐ and PreS‐specific IgG response which consists of an early IgG_1_ followed by a late but sustained IgG_4_ subclass response.^29^, ^30^, ^35^, ^42^ The late and sustained IgG_4_ response is considered to be responsible for the long‐term protective effect of allergen‐specific immunotherapy which persists for several years even after discontinuation of vaccination.^32^, ^35^, ^45^, ^46^

Figure 4D shows the development of RBD‐specific IgG subclass responses in the subject after immunization with folded HEK cell‐expressed PreS‐RBD. A strong IgG_1_ subclass response to folded RBD was observed after the second vaccination whereas folded RBD‐specific IgG_4_ antibodies increased only later, that is, after the third vaccination. Low RBD‐specific IgG_2_ levels and no RBD‐specific IgG_3_ responses were found (Figure 4D). In parallel, we analyzed S and RBD‐specific IgG responses in randomly enrolled healthy subjects approximately 4 weeks after full vaccination with SARS‐CoV‐2 vaccines registered in Europe (i.e., Janssen COVID‐19 vaccine, Johnson&Johnson; Vaxzevria, AstraZeneca; Comirnaty, BionTech/Pfizer) (Table S1, Figure S6). Four out of the nine randomly enrolled healthy vaccinated subjects (i.e., A287, A292, A077, and C019) mounted only low S‐ and almost no RBD‐specific IgG responses (Figure S6). The quantification of S1‐specific antibody levels confirmed these results, showing that they had S1‐specific antibody levels below 200 BAU/ml (Table 1) which were lower than those of the majority (i.e., eight out of ten) of COVID‐19 convalescent subjects (Table 1). The RBD‐specific IgG subclass response in subjects vaccinated with registered vaccines consisted mainly of an IgG_1_ subclass response, little IgG_2_, almost no IgG_4,_ and no IgG_3_ (Figure 4D).

**TABLE 1:** SARS‐CoV‐2‐specific protective antibodies in sera obtained at different time points from the subject, from COVID‐19‐convalescent patients and subjects after vaccination with registered SARS‐CoV‐2 vaccines

	Sample ID	% Inhibition^a^		Quantitative determination of S1‐specific IgG [BAU/ml]^b^	Neutralization assay [VNT50 titer]^c^	Virus neutralization test [titer]^d^
		100ng RBD	50ng RBD			
Immunization with						
PreS‐RBD [*E*.*coli*]	V1	6.8	−2.0	<3.2	<10	<20
	V2	−4.3	−2.1	<3.2	n.d.	<20
	V3	2.2	8.9	<3.2	n.d.	n.d.
PreS‐RBD [*E*.*coli*]	V4	−0.9	−8.4	<3.2	n.d.	n.d.
	V5	−3.0	5.0	<3.2	n.d.	n.d.
PreS‐RBD [*E*.*coli*]	V6	−0.9	−5.7	<3.2	<10	n.d.
	V7	0.2	−5.3	<3.2	n.d.	n.d.
	V8	3.9	−4.5	<3.2	<10	n.d.
PreS‐RBD [*HEK*]	V9	−8.5	1.7	<3.2	<10	<20
	V10	5.6	5.8	<3.2	<10	n.d.
	V11	8.6	6.6	<3.2	<10	n.d.
	V12	−4.3	5.2	42.8	<10	n.d.
PreS‐RBD [*HEK*]	V13	1.5	−6.0	59.7	<10	<20
	V14	48.1	90.5	1530.0	57	30
	V15	40.3	88.9	1653.4	87	60
	V16	46.2	95.9	1275.7	78	40
PreS‐RBD [*HEK*]	V17	94.2	81.4	1130.1	39	40
	V18	95.6	100.1	2360.0	171	120
	V19	83.9	96.5	2864.1	267	160
	V20	98.5	99.7	2745.2	209	120
Immunization with licensed SARS‐CoV‐2 vaccines						
Janssen COVID‐19 Vaccine	A077	5.1	5.1	130.2	25	10
Janssen COVID‐19 Vaccine	C019	1.2	−1.7	91.0	12	10
Vaxzevria	A287	35.1	32.5	157.3	18	10
Vaxzevria	A292	−8.6	−14.4	164.5	59	30
Comirnaty	A288	3.5	95.2	1309.6	166	120
Comirnaty	A286	97.3	98.6	2853.8	836	320
Comirnaty	A290	16.0	52.8	838.2	90	60
Comirnaty	A291	82.9	99.4	1414.4	207	120
Cross‐vaccination (Vaxzevria, Comirnaty)	A289	98.3	99.0	2728.9	522	240
	Median values^e^		**52.8**	**838.2**	**90**	**60**
COVID‐19 convalescent patients						
Mild COVID‐19	B001	−18.3	3.9	476.4	n.d.	n.d.
Mild COVID‐19	B002	−23.0	7.2	376.6	n.d.	n.d.
Mild COVID‐19	B013	56.5	87.6	149.6	n.d.	n.d.
Mild COVID‐19	B014	14.7	30.3	111.1	n.d.	n.d.
Mild COVID‐19	B015	46.0	99.8	910.9	n.d.	n.d.
Severe COVID‐19	I001	47.1	96.8	667.9	n.d.	n.d.
Severe COVID‐19	I002	9.6	19.5	1186.5	n.d.	n.d.
Severe COVID‐19	I003	19.8	25.8	2963.8	n.d.	n.d.
Severe COVID‐19	I005	26.8	99.7	2606.0	n.d.	n.d.
Severe COVID‐19	I007	76.8	100	859.9	n.d.	n.d.
	Median values^f^	**23.3**	**58.95**	**763.9**	**n.d.**	**n.d.**

Abbreviation: n.d., not done.

^a^ % Inhibition of RBD binding to ACE2 determined by molecular interaction assay with 100 or 50 ng RBD.

^b^ Quantitative determination of S1‐specific IgG performed with Anti‐SARS‐CoV‐2‐QuantiVac‐ELISA kit (Euroimmun,Lübeck, Germany). Results are calculated as WHO‐standardized binding antibody units (BAU)/ml and values >35.2 BAU/ml are considered as positive.

^c^ Virus neutralization given as VNT50 titers (50% virus neutralization titer) of plasma samples with 600 TCID_50_ SARS‐CoV‐2. Values <10 are considered as negative.

^d^ 100% Virus neutralization titers with 50–100 TCID_50_ SARS‐CoV‐2. Values <20 are considered as negative.

^e^ Median values subjects vaccinated with a registered SARS‐CoV‐2 vaccine (Table S1).

^f^ Median values of COVID‐19 convalescent patients.

### 3.6 Antibodies in serum, tears, and nasal secretions of the PreS‐RBD‐immunized subject recognize exclusively conformational RBD epitopes

A detailed analysis of the SARS‐CoV‐2‐specific antibody responses was performed in the immunized subject using a solid‐phase chip containing a large panel of micro‐arrayed SARS‐CoV‐2 proteins as well as peptides spanning the S protein^24^ during the entire period of immunization with folded and unfolded PreS‐RBD (V1‐V20) (Figure S7). Immunization with unfolded *E*. *coli*‐expressed PreS‐RBD only induced antibodies against the immunogen (i.e., unfolded *E*. *coli*‐expressed PreS‐RBD) but no SARS‐CoV‐2‐specific IgG, IgA, or IgM antibody response (Figure S7A–C, visits 1–9), which is accordance with our previous study.^24^ Immunization with folded HEK cell‐expressed PreS‐RBD induced a strong and sustained IgG response against folded RBD and against proteins containing folded RBD (i.e., insect cell‐expressed S and S1, HEK cell‐expressed S1) (Figure S7A, left part) whereas no IgG responses to sequential RBD‐derived peptide epitopes were detected (Figure S7A, right part). The RBD‐specific IgG response was accompanied by an initially strong but only transient IgA response specific for folded RBD (Figure S7B). No relevant SARS‐CoV‐2‐specific IgM responses were found throughout the immunization period (Figure S7C). Immunization with folded PreS‐RBD boosted the IgG response against unfolded PreS‐RBD which is attributable to PreS‐specific IgG antibodies (Figure S7A). Of note, no antibodies specific for the nucleocapsid protein (NP) or for S2 lacking RBD were observed during the whole period of immunization and observation (Figure S7A–C). Figure S8A‐C shows that high levels of IgG antibodies specific for folded RBD and to a lower extent to *E*.*coli*‐expressed unfolded PreS‐RBD were also present in nasal secretions and tears obtained at visits 15 and 18. At these time points, also moderate levels of IgA antibodies specific for folded RBD could be detected whereas no SARS‐CoV‐2‐specific IgM antibodies were found (Figure S8B,C).

### 3.7 SARS‐CoV‐2‐specific antibody responses in the subject immunized with PreS‐RBD are accompanied by cellular responses

Table S2 provides an overview of the analysis of cellular immune responses during the immunization period (visits 1–20). Approximately 1 week after immunization with unfolded PreS‐RBD (V2 and V5), we observed a doubling of the percentages of plasmablasts (Figure S9). This increase of the percentage of plasmablasts was extremely strong 1 week after the first immunization with folded PreS‐RBD at visit 11 when 13.1% were detected. Almost a doubling of plasmablasts was also observed 1 week after the second (i.e., visit 14) and the third (i.e., visit 18) immunization (Table S2, Figure S9). The plasmablasts showed mainly the phenotype CD19^+^IgM‐CD38^++^ CD21^−^ and CD10^−^ that had been observed also in another vaccination study.^47^ The strong increase of plasmablasts 1 week after the first immunization with folded HEK cell‐expressed PreS‐RBD (Table S2, visit 11) was accompanied by an increase of CD21^−^ B cells (mainly due to the increase of IgM‐CD38^++^ plasmablasts) as well as of CD21^−^CD10^−^ hyper‐activated B cells. Marked increases in central memory CD4^+^ and CD8^+^ T cells (CD45RO^+^CCR7^+^) were observed only 1 week after the first immunization with unfolded *E*. *coli*‐expressed (i.e., visit 2) and 1 week after the first immunization with folded HEK cell‐expressed PreS‐RBD (i.e., visit 11) (Table S2).

At visit 9, when the first immunization with folded PreS‐RBD was performed no RBD and PreS‐specific CD4^+^ and CD8^+^ T‐cell responses were detected (Figure S10A). They became only detectable after immunization with folded PreS‐RBD at visits 11, 14, 17, and 19. The RBD‐specific CD4^+^ and CD8^+^ T‐cell responses had a similar magnitude as those observed in subjects vaccinated with registered SARS‐CoV‐2 vaccines (Figure S10A,B). Of note, RBD‐specific T‐cell responses were not always associated with RBD‐specific IgG antibody responses. For example, subjects A286 and A291 had no relevant RBD‐specific T‐cell responses but had RBD‐specific IgG antibodies and subject A077 showed RBD‐specific T‐cell responses but had no relevant RBD‐specific IgG antibodies (Figures S6 and S10).

### 3.8 Antibodies induced by immunization of the subject with folded PreS‐RBD inhibit binding of RBD to ACE2 and neutralize SARS‐CoV‐2

Table 1 provides an overview of the development of S1‐specific IgG antibodies, of antibodies inhibiting the binding of RBD to ACE2 and of virus‐neutralizing antibodies in the immunized subject. Sera obtained from nine healthy subjects approximately 4 weeks (median = 27 days) after full vaccination with licensed genetic COVID‐19 vaccines (Table S1) and sera from ten COVID‐19 convalescent patients obtained approximately 8 weeks after SARS‐CoV‐2 infection were included in the assays for comparison. Immunization with unfolded *E*. *coli*‐expressed PreS‐RBD neither induced S1‐specific IgG antibodies nor antibodies inhibiting the interaction of RBD and ACE2, and also, no virus‐neutralizing antibodies were detected (Table 1, visits 1–9). At visits 19 and 20 (i.e., 3 and 4 weeks after the third immunization), S1‐specific IgG antibody levels exceeded 2700 BAU/ml in the PreS‐RBD‐immunized subject and were higher than the median S1‐specific IgG antibodies in subjects vaccinated with licensed vaccines (i.e., 91.0–2853.8 BAU/ml; median: 838.2 BAU/ml) and in COVID‐19 convalescent subjects (i.e., 111.1–2963.8 BAU/ml; median: 763.9 BAU/ml). Serum obtained from the immunized subject at visit 20 inhibited the binding of 100 and 50 ng RBD to ACE2 by more than 98% whereas median inhibitions obtained with sera from subjects vaccinated with licensed genetic vaccines (100 ng RBD: −8.6% to 98.3% inhibition, median inhibition: 16.0%; 50 ng RBD: −14.4% to 99.4% inhibition, median inhibition: 52.8%) and sera from COVID‐19 convalescent subjects were much lower. In the virus neutralization assay using 600 TCID_50_ authentic SARS‐CoV‐2 (BetaCoV/Munich/BavPat1/2020 isolate), the VNT50 titer (which indicates the reciprocal serum dilution yielding a 50% reduction in anti‐SARS‐CoV‐2 NP staining of the infected Vero cells measured by ELISA 2 days later) of the PreS‐RBD‐immunized subject at visit 19 and 20 was 267 and 209, respectively, was higher than the median VNT50 titers (i.e., 12–839; median: 90) found for subjects vaccinated with licensed vaccines. These results are in agreement with the data obtained by the virus neutralization test using 50–100 TCID_50_ SARS‐CoV‐2, which expresses the VNT as reciprocal serum dilution required for 100% protection against virus‐induced cytopathic effects. The VNTs in this assay obtained for the PreS‐RBD‐vaccinated subject at visits 19 and 20 were 160 and 120, respectively, and thus also higher than the median VNT (10–320; median: 60) as determined in subjects vaccinated with licensed vaccines.

Figure S11 shows the correlations between antibody levels specific for S1, and RBD, inhibitions of RBD binding to ACE2 and virus neutralization titers for those samples for which data pairs are available in Table 1. Highly significant correlations were found for S1‐ and RBD‐specific IgG (Figure S11A), S1‐specific IgG and percentages inhibition of RBD to ACE2 binding (Figure S11B), VNT50 titers and percentages inhibition of RBD to ACE2 binding (Figure S11C) and percentages inhibition of RBD to ACE2 binding and VNTs (Figure S11D).

## 4 DISCUSSION

The first generation of COVID‐19 vaccines introduced for global application mainly comprised genetic vaccines, inactivated whole virus vaccines but only one subunit vaccine which is in the final stage of receiving authorization^1^, ^5^ (https://www.who.int/publications/m/item/draft‐landscape‐of‐covid‐19‐candidate‐vaccines). Available data for those vaccines which are used in mass immunization programs worldwide suggest that COVID‐19 vaccines will indeed help to control the pandemic and reduce SARS‐CoV‐2 associated deaths. However, the number of SARS‐CoV‐2 infections is steadily increasing, indicating that the virus is becoming endemic. The SARS‐CoV‐2 subunit vaccine PreS‐RBD reported by us here has several features, which may make it an attractive candidate vaccine addressing important needs in the field of COVID‐19 vaccination that are not yet completely met by currently available SARS‐CoV‐2 vaccines.

Our recombinant PreS‐RBD fusion protein can be produced in large quantities and high purity through expression in mammalian cells such as HEK cells which is a process that is well established all over the world not only for vaccines but also for the production of antibodies and biologics. Furthermore, HEK cells are able to express folded proteins with posttranslational modifications such as N‐glycosylation as they occur in the natural protein. As previously reported,^24^ we demonstrate that it is important to obtain the immunogen/antigen as a structurally folded protein because immunization with unfolded PreS‐RBD failed to induce RBD‐specific antibodies that are necessary to inhibit the RBD‐ACE2 interaction and to achieve virus neutralization. We show here that the determination of the structural fold of PreS‐RBD can be performed by using biophysical methods such as circular dichroism (CD) spectroscopy analysis of the protein and/or by showing the reactivity of the recombinant antigen with IgG antibodies from COVID‐19 convalescent patients which specifically react with the folded but not with the unfolded, *E*‐*coli*‐expressed PreS‐RBD (Figure 1C,G).

PreS‐RBD is a recombinant protein, and thus, it is possible to perform precise dose‐finding studies to determine the optimal amount of the immunogen for vaccination which is not possible for genetic vaccines.^5^ In fact, it has been reported that the amount and quality of S protein produced upon genetic vaccination may vary considerable between the different genetic vaccines and depending on delivery of the S‐encoding genetic information into the host cells.^5^ S antigen produced after genetic vaccination does not only remain at the injection site but may become expressed at sites and in cells distant to the injection site and may even circulate in the vaccinated subject and via adherence to ACE2 in the circulation may contribute to some of the observed rare side effects associated with vaccination. We have formulated the recombinant PreS‐RBD by adsorption to aluminum hydroxide, an adjuvant which has been safely used in numerous vaccines for decades. In our pilot stability studies, we found that approximately 90% of PreS‐RBD is bound to aluminum hydroxide and thus the injected antigen remains to a large extent at the injection site (Gattinger and Valenta, unpublished). Importantly, aluminum hydroxide‐formulated PreS‐RBD remains stable for months at +4°C and also storage at higher temperature does not seem to affect the stability and immunogenicity of the vaccine which is important for a vaccine to be distributed and used globally, especially in countries with low resources (data not shown).

Our study indicates that administration of two to three doses of a molar equivalent of 40 μg of folded PreS‐RBD induces a robust induction of RBD‐specific antibody responses, which are accompanied by specific T‐cell responses and induction of B memory/plasma cell responses. Results obtained in the herein immunized subject demonstrate that the RBD‐specific antibody response consists mainly of an IgG response composed of an early IgG_1_ and late IgG_4_ response of which the latter was not observed with genetic COVID‐19 vaccines (Figure 4D) so far. The biphasic induction of RBD‐specific (i.e., early IgG_1_ and late, sustained IgG_4_) is very similar to that of BM32, a therapeutic grass pollen allergy vaccine which contains recombinant fusion proteins consisting of PreS and allergen peptides.^35^ BM32 has been safely used for the treatment of grass pollen induced allergy in several clinical studies^41^, ^42^ (ClinicalTrials.gov Identifier: NCT02643641) and it has been shown that BM32‐induced PreS‐specific antibodies protect against HBV infections *in vitro* because they are directed against the N‐terminal part of PreS containing the binding site of HBV for the NTCP receptor on human hepatocytes.^29^, ^30^ In fact, very recently, it has been shown that immunization of patients with chronic HBV infections with BM325 (i.e., VVX001) had induced HBV‐neutralizing antibodies *in vivo*.^43^ Furthermore, PreS‐RBD not only induced RBD‐specific IgG antibodies but also PreS‐specific antibodies reacting with the NTCP binding sites of HBV genotypes A‐H and hence may protect also against HBV infections (Figure 4C, Figures S4 and S5). However, the PreS‐RBD fusion protein was made by us not only with the intention to induce SARS‐CoV‐2‐ and HBV‐neutralizing antibodies but to use PreS also as a carrier protein to enhance the immunogenicity of RBD. In a previous study, we found that approximately 20% of SARS‐CoV‐2‐infected patients did not produce RBD‐specific IgG antibodies.^23^, ^24^ Further studies are needed to investigate the reasons for RBD‐unresponsiveness which may include genetic factors such as HLA‐association of RBD‐specific immune responses and/or different repertoires of specific T cells and B cells. RBD‐specific antibodies are important for the induction of sterilizing immunity to SARS‐CoV‐2 because these antibodies prevent the virus from binding to its receptor ACE2 on human cells and thus are critically important for virus neutralization.^22^, ^23^, ^24^ We therefore hypothesized that immunization with RBD alone will eventually not be sufficient to induce uniform and robust RBD‐specific antibodies in an outbred population. Indeed, our hypothesis was supported by results obtained from the immunization of outbred rabbits with RBD alone and the PreS‐RBD fusion protein. In this study and in a previous study,^24^ we found that approximately 20%–30% of rabbits failed to produce robust RBD‐specific antibodies when they were immunized with RBD alone whereas all rabbits immunized with PreS‐RBD produced uniform and robust RBD‐specific antibodies. This result can be explained by the hapten‐carrier principle discovered by the Nobel laureate Benacerraf, who demonstrated that covalent coupling or fusion of a less immunogenic component (i.e., the hapten) to a protein carrier can enhance the immunogenicity of the hapten.^31^ We used this principle extensively for the construction of allergy vaccines based on allergen‐derived peptides fused to PreS to enhance the immunogenicity of the allergen peptides.^38^ Thus, the results obtained in this study are in agreement with our previous work performed in AIT. However, we noted an important difference between the PreS‐based SARS‐CoV‐2 subunit‐vaccine described in our study and previously described AIT vaccines based on PreS‐fusion proteins. In order to induce antibody responses against folded RBD capable of neutralizing SARS‐CoV‐2, it was necessary to express PreS‐RBD in eukaryotic cells, in particular in mammalian cells as folded protein whereas PreS‐RBD expressed in *E*. *coli* as unfolded protein did not induce antibodies recognizing folded RBD which were capable of neutralizing SARS‐CoV‐2. By contrast, AIT vaccines based on unfolded PreS‐fusion proteins induced IgG antibodies capable of recognizing folded natural allergens and preventing allergic patients IgE binding.^38^, ^48^

Whether a vaccine based on PreS‐RBD will be able to overcome RBD‐non‐responsiveness needs of course to be demonstrated in extensive vaccination trials. However, our initial data encourage to move into this direction.

The RBD‐specific IgG antibodies induced in the human subject with PreS‐RBD cross‐reacted with RBD mutants and variants including even the highly mutated VOC omicron (Figure 4B, Figures S1–S3)^17^, ^18^, ^19^, ^20^ suggesting that the PreS‐RBD‐based vaccine has the potential to cross‐protect even against strongly mutated VOCs. PreS‐RBD contains two RBD domains, one fused to the N‐ and one fused to the C‐terminus of PreS, and it is therefore be quite easy to enhance the cross‐protective effect by including RBDs from the two most divergent and most common SARS‐CoV‐2 VOCs in the PreS‐RBD construct. This would have the advantage that the relevant epitopes of two SARS‐CoV‐2 VOCs can be included in only one antigen, which will allow addressing the challenge of emerging virus variants in a highly effective manner.

The RBD‐specific antibodies induced in the PreS‐RBD‐immunized subject were found to block more strongly the binding of RBD to ACE2 than those obtained from subjects after full vaccination with currently available and licensed COVID‐19 vaccines and from COVID‐19 convalescent patients when determined by their median blocking activity (Table 1). These results were confirmed by testing the VNTs using two different virus neutralization assays, one measuring the production of virus antigen and the second determining the virus cytopathic effect.

In addition to the fact that folded PreS‐RBD induces antibodies which block RBD‐ACE2 binding and thus infection of the host cell, also other observations indicate, that the folded PreS‐RBD has features of a vaccine which could be used to induce sterilizing immunity against SARS‐CoV‐2 infections. One of these observations is that the RBD‐specific antibodies were not only detected in serum but also in mucosal fluids (i.e., tear and nasal fluids) which are derived from the sites where the virus initially enters the body, infects host cells and initially replicates. A similar finding was obtained also for AIT vaccines which in fact block the docking of allergens to IgE antibodies bound to the effector cells of allergy at mucosal sites and thus prevent local allergic inflammation.^49^, ^50^

Another important finding was that immunization with PreS‐RBD induced not only a first short‐lived wave of specific IgG_1_ antibodies but also a second wave of late but sustained IgG_4_ antibodies. In fact, it is known from AIT that AIT‐induced allergen‐specific IgG_4_ antibodies persist in vaccinated subjects for a long time and are therefore considered to be important for the long‐term protective effect of AIT even after discontinuation of treatment.^32^, ^46^ Thus, PreS‐RBD may have the potential to induce long‐lasting sterilizing immunity against SARS‐CoV‐2 via induction of sustained production of RBD‐specific IgG_4_ antibodies which actually are considered as non‐inflammatory neutralizing antibodies.^51^

Finally, we would like to comment on the safety of the PreS‐RBD‐based subunit vaccine. It is of course a limitation of this study that we do yet have data from extensive toxicity studies or vaccination trials in humans which will be the focus of next clinical studies. However, it should be mentioned that there were no adverse events observed in the immunized rabbits of which each has received so far five doses of the vaccine. There were also no adverse side effects observed in the immunized subject. However, one may consider the huge experience with aluminum hydroxide‐formulated vaccines which have been used safely for decades. In particular in AIT, aluminum‐adsorbed vaccines are given often more than twenty times per year for several years^32^ and the PreS‐based allergy vaccine BM32 has been used safely extensively in clinical AIT trials^29^, ^30^, ^35^, ^41^, ^42^ and also for vaccination against HBV.^43^

In summary, we report the *in vitro* and *in vivo* characterization of a SARS‐CoV‐2 subunit vaccine which seems to have the potential of inducing sterilizing immunity to SARS‐CoV‐2 variants.

## CONFLICT OF INTEREST

Rudolf Valenta has received research grants from HVD Life‐Sciences, Vienna, Austria, WORG Pharmaceuticals, Hangzhou, China and from Viravaxx AG, Vienna, Austria. He serves as consultant for Viravaxx AG. Rudolf Valenta, Pia Gattinger, Bernhard Kratzer, and Winfried Pickl are authors on a patent application regarding the vaccine. The other authors have no conflict of interest to declare.

## AUTHOR CONTRIBUTIONS

PG designed and performed experiments, analyzed data, wrote manuscript, and read manuscript. BK, ITu, AOR, LG, KB, KN, EGS, and DT performed experiments, analyzed data, and read manuscript. GH, WK, KM, AK, HS, ITa, UW, and WFP analyzed data and read manuscript. RV designed and supervised experiments, performed self‐immunization, analyzed data, wrote manuscript, and read manuscript.

## Supporting information

Fig S1‐Fig S11

Supplementary Material
