# Supplementary material for: Vaccine based on folded RBD‐PreS fusion protein with potential to induce sterilizing immunity to SARS‐CoV‐2 variants
Source: Allergy. 2022 Apr 15:10.1111/all.15305. Online ahead of print. doi: 10.1111/all.15305 (PMC9111473; doi:10.1111/all.15305)
Supplement: Supplementary file 1 — Fig S1‐Fig S11 [file ALL-9999-0-s002.pdf]

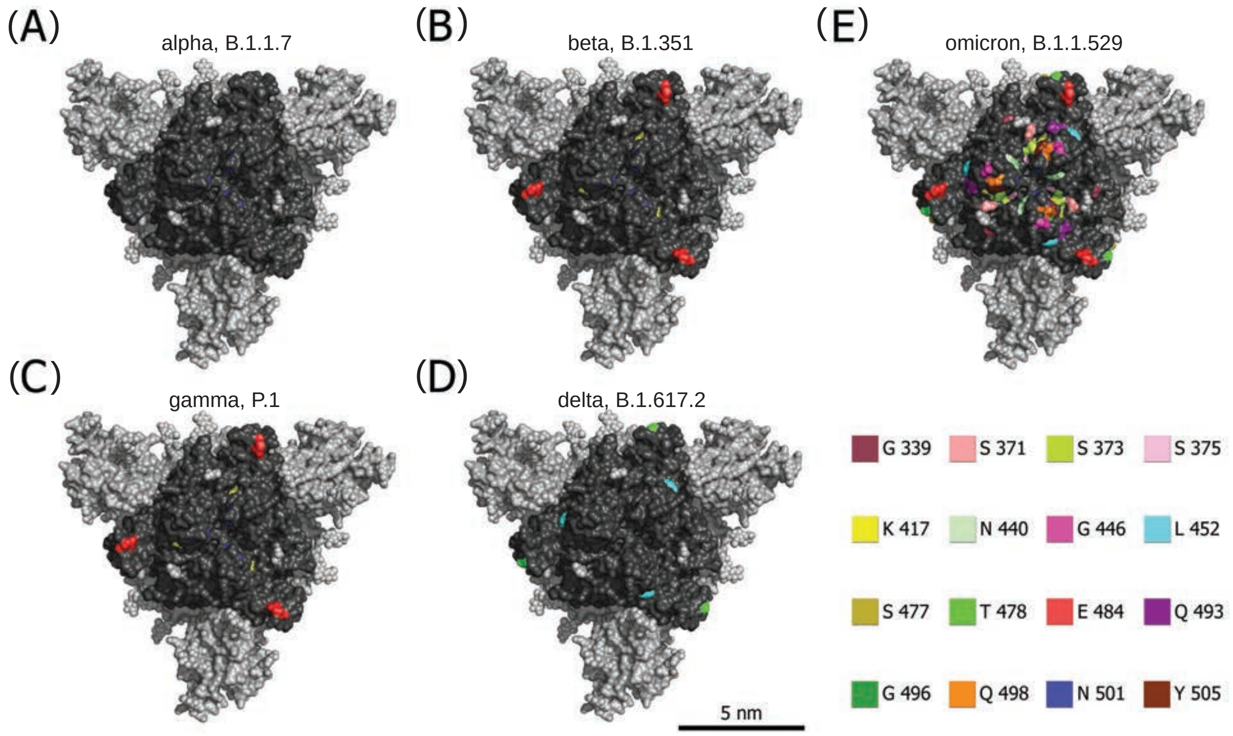

Figure S1_Gattinger et al.

```
Wuhan 330   PNITNLCPFGEVFNATRFASVYAWNRKRISNCVADYSVLYNSASFSTFKCYGVSPTKLND 390
Alpha 330   ............................................................ 390
Beta  330   ............................................................ 390
Gamma 330   ............................................................ 390
Delta 330   ............................................................ 390
Omcrn 330   .........D...............................L.P.F.............. 390

Wuhan 391   LCFTNVYADSFVIRGDEVRQIAPGQTGKIADYNYKLPDDFTGCVIAWNSNNLDSKVGGNY 450
Alpha 391   ............................................................ 450
Beta  391   ..........................N................................. 450
Gamma 391   ..........................T................................. 450
Delta 391   ............................................................ 450
Omcrn 391   ..........................N......................K.....S.... 450

Wuhan 451   NYLYRLFRKSNLKPFERDISTEIYQAGSTPCNGVEGFNCYFPLQSYGFQPTNGVGYQPYR 510
Alpha 451   ..................................................Y......... 510
Beta  451   .................................K................Y......... 510
Gamma 451   .................................K................Y......... 510
Delta 451   .R.........................K................................ 510
Omcrn 451   ..........................NK.....A........R..S.R..Y...H..... 510

Wuhan   511   VVVLSFELLHAP   522
Alpha   511   ............   522
Beta    511   ............   522
Gamma   511   ............   522
Delta   511   ............   522
Omcrn   511  . ...........   522
```

[1]Sequence variants alpha (Pango B.1.1.7), beta (Pango B.1.351/B.1.351.2/B.1.351.3), gamma (Pango P.1/ P.1.1/ P.1.2), delta (Pango B.1.617.2) and omicron (omcrn) (Pango B.1.1.529).

Figure S2_Gattinger et al.

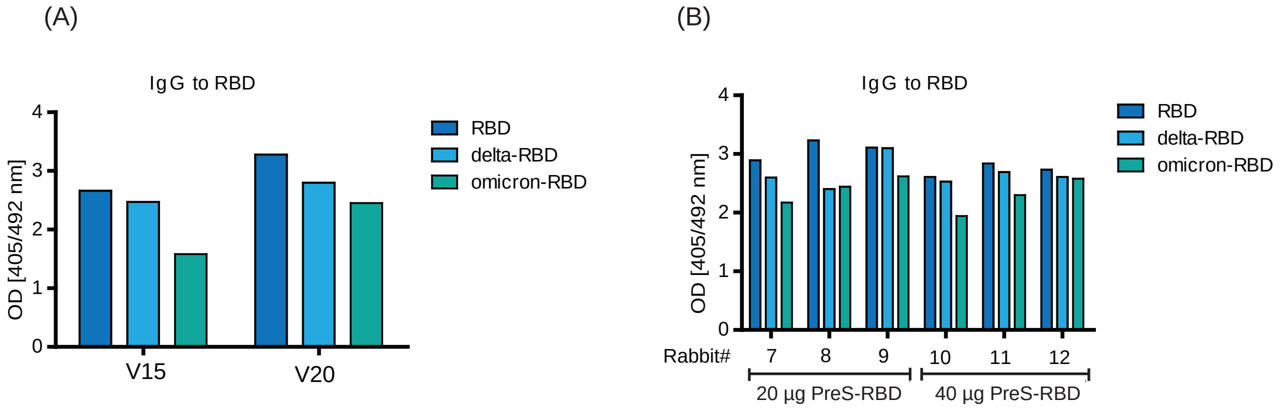

Figure S3_Gattinger et al.

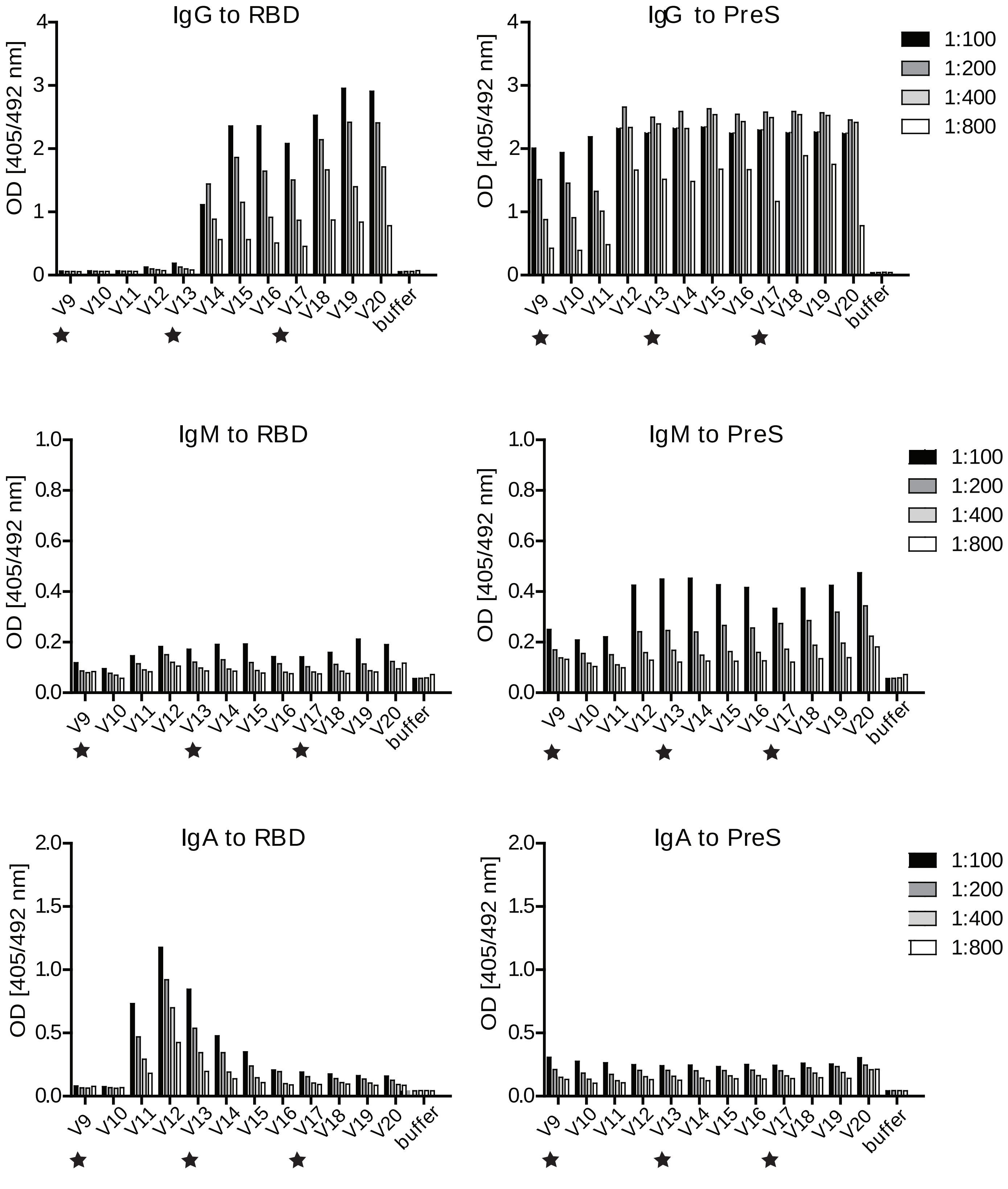

Figure S4_Gattinger et al.

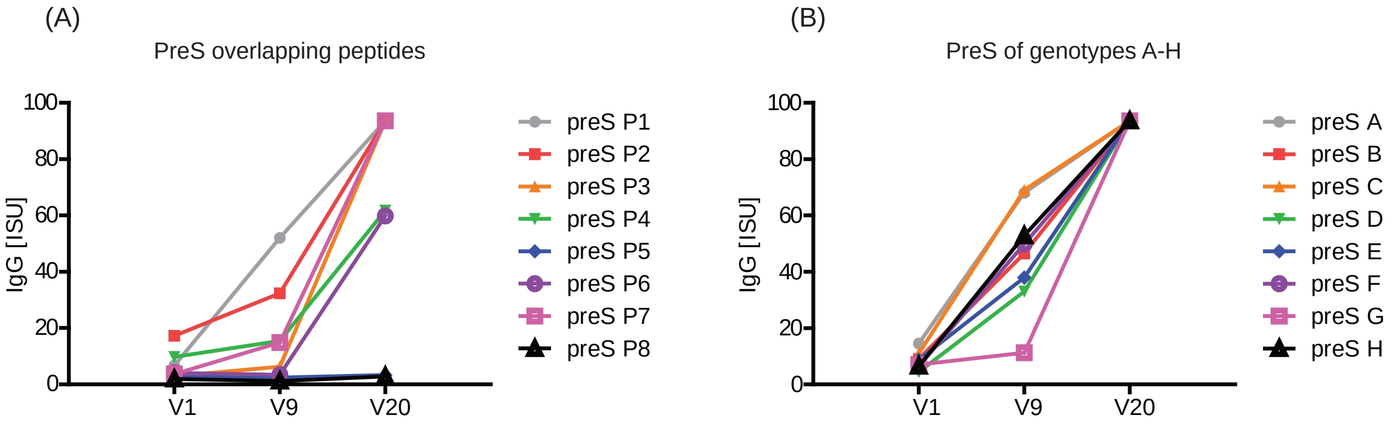

Figure S5_Gattinger et al.

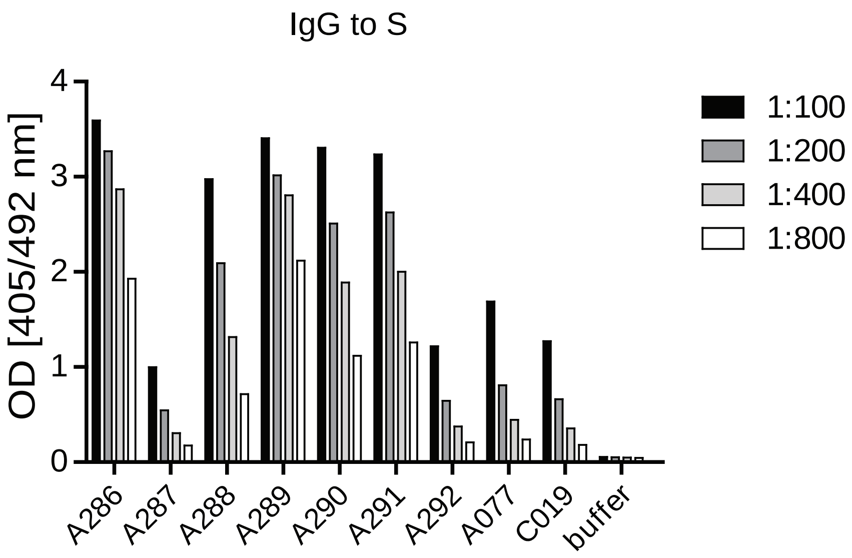

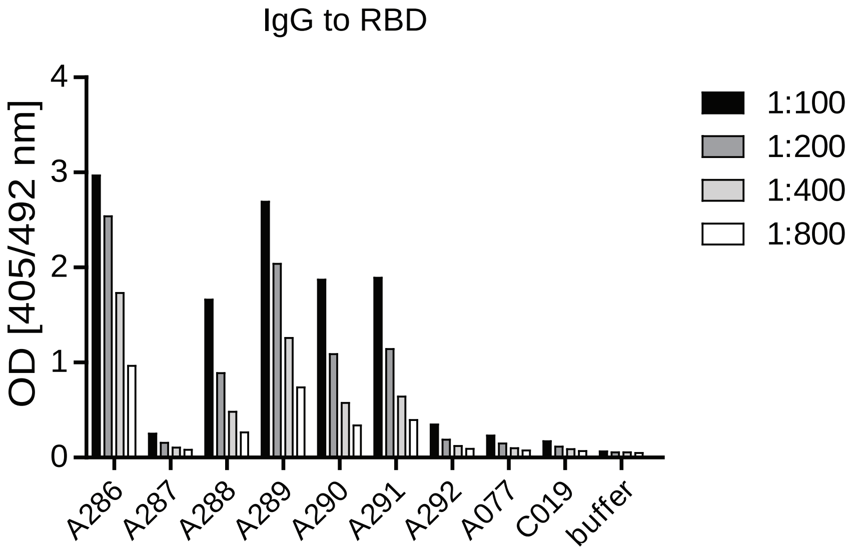

Figure S6_Gattinger et al.

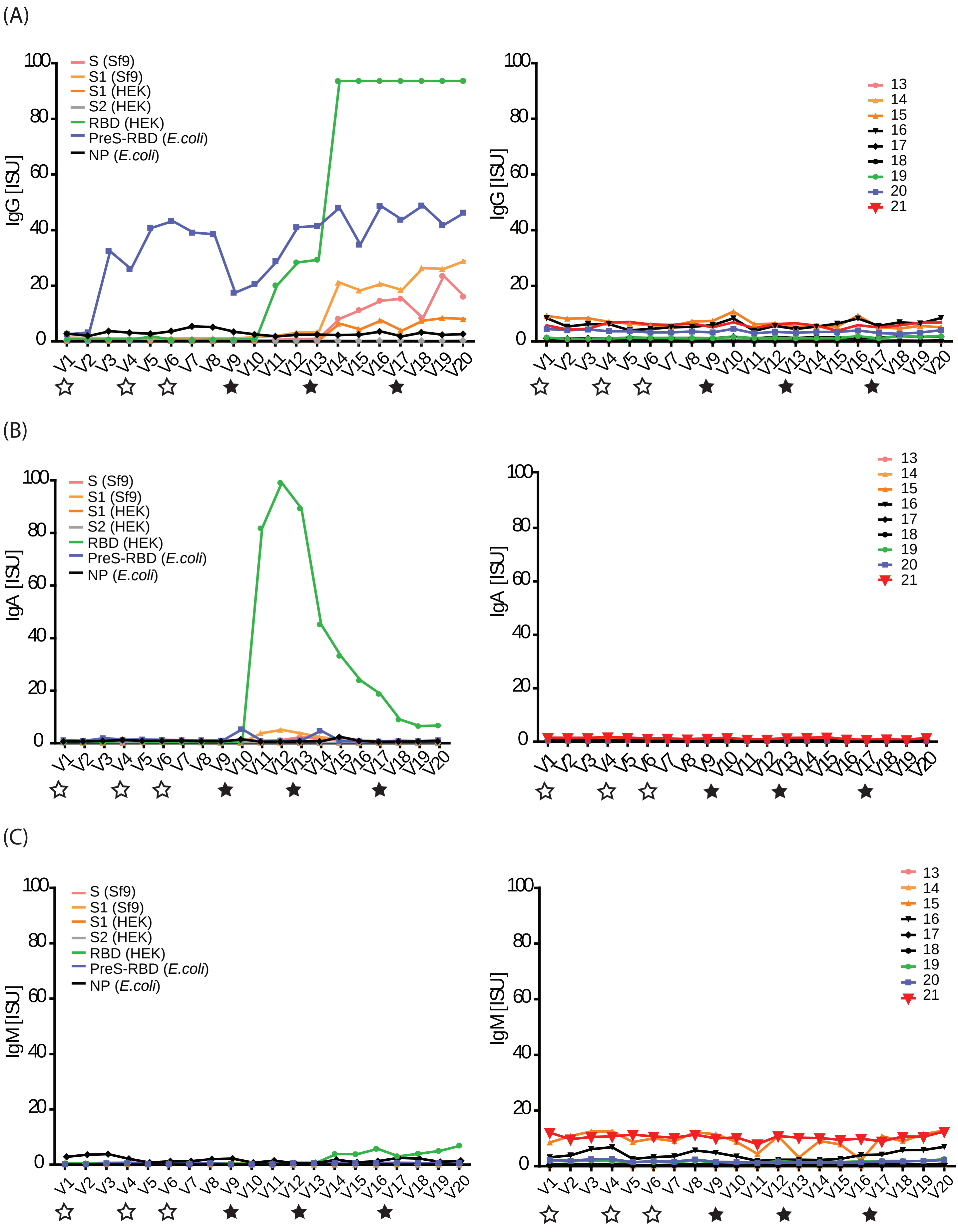

Figure S7_Gattinger et al.

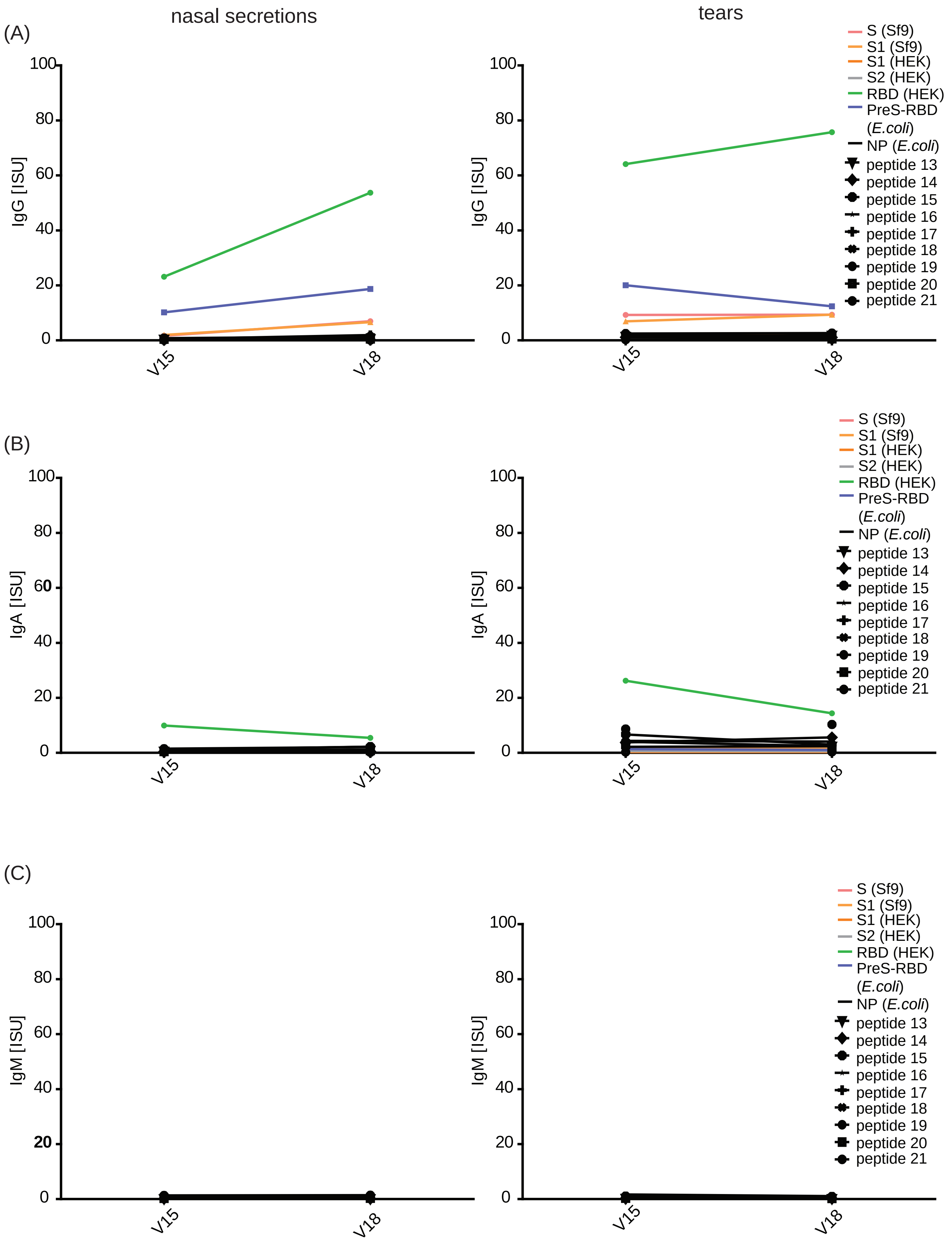

Figure S8_Gattinger et al.

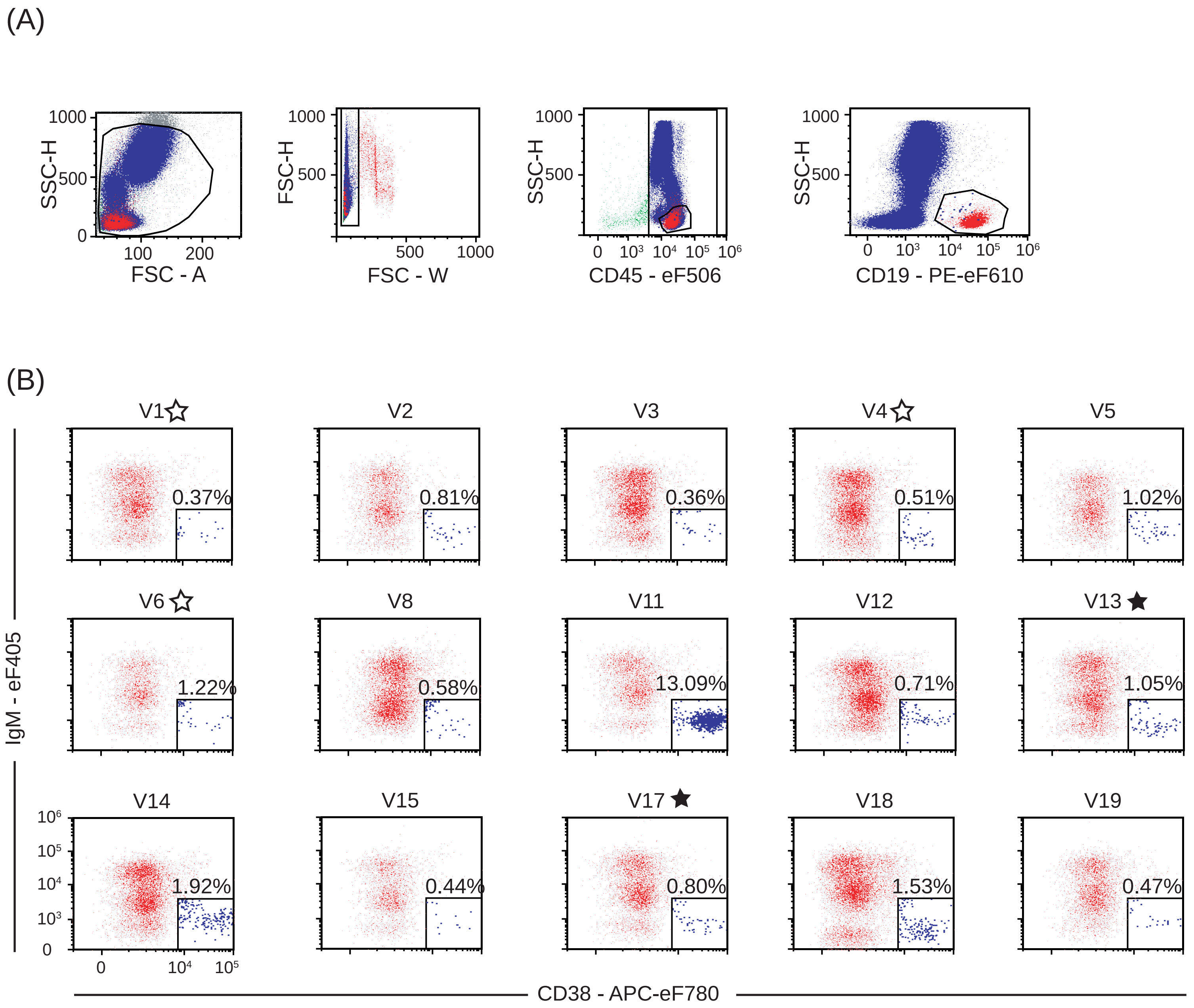

Figure S9_Gattinger et al.

(A)

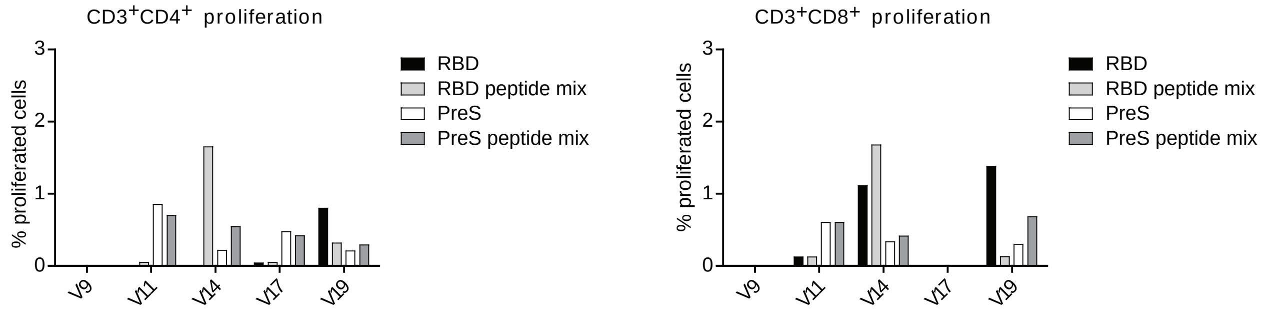

(B)

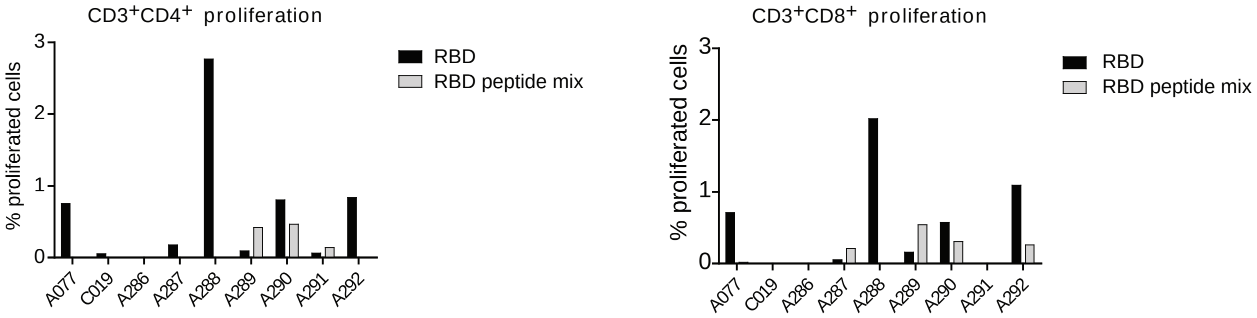

Figure S10_Gattinger et al.

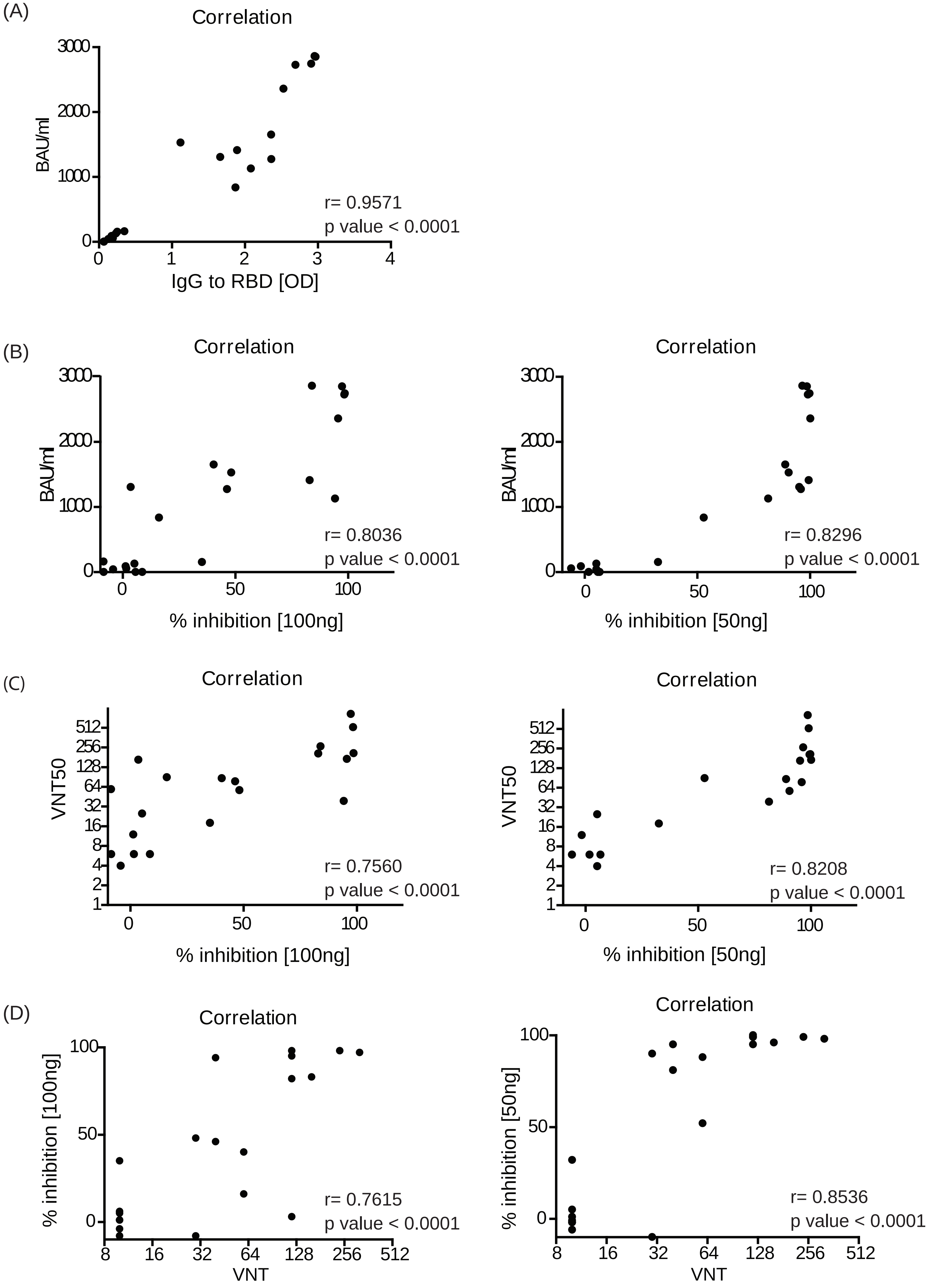

Figure S11_Gattinger et al.
