# Supplementary material for: Vaccine based on folded RBD‐PreS fusion protein with potential to induce sterilizing immunity to SARS‐CoV‐2 variants
Source: Allergy. 2022 Apr 15:10.1111/all.15305. Online ahead of print. doi: 10.1111/all.15305 (PMC9111473; doi:10.1111/all.15305)
Supplement: Supplementary file 2 — Supplementary Material [file ALL-9999-0-s001.docx]

**Appendix: Online Repository**

**Vaccine based on folded RBD-PreS fusion protein with potential to induce sterilizing immunity to SARS-CoV-2 variants**

Pia Gattinger, PhD^a^, Bernhard Kratzer, PhD^b^, Inna Tulaeva, MD, PhD^a,c^ , Katarzyna Niespodziana, PhD^a,d^, Anna Ohradanova-Repic, PhD^e^, Laura Gebetsberger, MSc^e^, Kristina Borochova, PhD^a^, Erika Garner-Spitzer, PhD^f^, Doris Trapin, MSc^b^, Gerhard Hofer, PhD^g^, Walter Keller, PhD^h^, Isabella Baumgartner, MD^i^, Ivan Tancevski, MD^j^, Musa Khaitov, MD^k,l^, Alexander Karaulov, MD^c^, Hannes Stockinger, PhD^e^, Ursula Wiedermann, MD, PhD^f^, Winfried F. Pickl, MD^b,d^, Rudolf Valenta, MD^a,c,d,k*^

^a^Department of Pathophysiology and Allergy Research, Division of Immunopathology, Center for Pathophysiology, Infectiology and Immunology, Medical University of Vienna, Vienna, Austria

^b^Institute of Immunology, Center for Pathophysiology, Infectiology and Immunology, Medical University of Vienna, Vienna, Austria

^c^Laboratory for Immunopathology, Department of Clinical Immunology and Allergology, Sechenov First Moscow State Medical University, Moscow, Russia

^d^Karl Landsteiner University of Health Sciences, Krems, Austria

^e^Institute for Hygiene and Applied Immunology, Center for Pathophysiology, Infectiology and Immunology, Medical University of Vienna, Vienna, Austria

^f^Institute of Specific Prophylaxis and Tropical Medicine, Medical University of Vienna, Vienna, Austria

^g^Department of Materials and Environmental Chemistry, University of Stockholm, Stockholm, Sweden

^h^Institute of Molecular Biosciences, BioTechMed Graz, University of Graz, Graz, Austria

^i^Department of Ophthalmology, Medical University Vienna, Vienna, Austria

^j^Department of Internal Medicine II, Medical University of Innsbruck, Innsbruck, Austria

^k^NRC Institute of Immunology FMBA of Russia, Moscow, Russia

^l^Pirogov Russian National Research Medical University, Moscow, Russia

* Corresponding author

Rudolf Valenta

Department of Pathophysiology and Allergy Research

Medical University of Vienna

Waehringer Guertel 18-20

A-1090 Vienna, Austria

Tel: +43-1-40400-50420

Tel: +43-69912570519

Fax: +43-1-40400-51300

E-mail: rudolf.valenta@meduniwien.ac.at

**Materials and Methods**

**Expression and purification of SARS-CoV-2-derived RBD and PreS-RBD**

For expression of the PreS-RBD fusion protein (Figure 1A) in mammalian cells a DNA encoding a fusion protein consisting of PreS ^37^ and SARS-CoV-2-derived RBD (aa330-aa522, SARS-CoV-2 Genbank accession Nr.: QHD43416.1) linked to the N- and C-terminus was prepared as synthetic gene. Likewise, a DNA coding only for SARS-CoV-2-derived RBD was made. The synthetic DNA molecules were codon optimized for the expression in HEK cells and contained a 5´ DNA coding for a N-terminal IL-2 signal peptide (MYRMQLLSCIALSLALVTNS) and a 3´ DNA coding for a C-terminal hexahistidine tag. The synthetic genes were cloned in frame into the mammalian expression vector pcDNA3.1(+) (Genscript, Leiden, Netherlands). Expi293F HEK cells (Thermofisher, Thermo-Fisher Scientific, Waltham, MA, USA) were grown in suspension in serum-free Expi293F expression medium (ThermoFisher) at 37°C, 85% relative humidity and 8% CO_2_ and 120 rpm according to the manufacturer’s instruction. Cells were split to a density of 2.5 x10^6^ cells/ml the day before transfection. Transfection was performed by mixing 25 µg plasmid DNA in 1.5 ml serum-free Opti-MEM medium (ThermoFisher) with 80 µl ExpiTransfectamine (ThermoFisher) in 1.5 ml Opti-MEM medium, incubation for 15 minutes at RT before dropwise adding to 25 ml Expi293F cells (density 3x10^6^ cells/ml). Cells were then incubated at described above. After 4 days of expression the cells were harvested by centrifugation for 10 min at 12000 rpm at 4°C and the supernatant was dialyzed (50 mM NaH_2_PO_4_, 300 mM NaCl, and 20 mM imidazole, pH 7.5) and then added to 2 ml Ni-NTA agarose (Qiagen, Hilden, Germany) over night at 4°C. Ni-NTA agarose was washed with 30 ml 50mM NaH_2_PO_4_, 300 mM NaCl, and 20 mM imidazole, pH 7.5 and bound protein was eluted stepwise with 50 mM NaH_2_PO_4_, 300 mM NaCl, and 250 mM imidazole, pH 7.5 in 2 ml fractions. Fractions were analyzed with Coomassie-stained SDS-PAGE and three 2ml fractions containing the majority of pure recombinant proteins were pooled and dialyzed against 50mM NaH_2_PO_4_, pH 7.0. The expression and purification of SARS-CoV-2 derived RBD and RBD-PreS-RBD fusion construct in *E.coli* was performed as previously described ^24^. Protein concentrations in the different protein preparations were determined before use with Pierce BCA Protein Assay Kit (Thermofisher) according to manufacturer’s instructions. Recombinant RBD variant proteins including alpha, beta, gamma, delta and omicron (Table S1, Figure S1) were obtained from Sino Biological Inc., Beijing, P.R.China. Mutations in the RBDs of the variants were shown in the sequence aligment (Table S1) visualized as surface representation of the SARS-CoV-2 spike protein trimer generated with PyMOL (PyMOL Molecular Graphics System, Version 2.5.0a0, Schrödinger, LLC) using PDB entry 6XR8 in Figure S1.

**Patient’s sera and ethics statement**

Serum samples from 10 convalescent COVID-19 patients with mild (n=5) and severe (n=5) symptoms obtained approximately 8 weeks after SARS-CoV-2 infection as confirmed by RT-PCR test and ten historic control sera ^24^ were used for the immunological characterization of recombinant proteins. Furthermore, a random sample of nine healthy subjects without history of SARS-CoV-2 infection who had been vaccinated with European Medicines Agency (EMA)-authorized COVID-19 vaccines were enrolled and blood sampling was performed four weeks after administration of two doses of Comirnaty (Pfizer/BionTech) (n=4) and Vaxzevria (AstraZeneca) (n=2) or one dose of Janssen COVID-19 vaccine (Johnson & Johnson) (n=2). One subject was cross-vaccinated with Vaxzevria and Comirnaty. Side effects occurring in these subjects after vaccination were assessed by questionnaire. Demographic and clinical data are presented in Table S2. All subjects provided written informed consent and experiments were approved by the Ethics Committees of the Medical University of Vienna (EK No.: 1302/2020).

**Biochemical, biophysical and immunological characterization of recombinant forms of RBD and PreS-RBD**

Purified recombinant proteins were assessed by SDS-PAGE followed by Coomassie blue staining under reducing and non-reducing conditions. Far UV circular dichroism (CD) spectra of RBD and PreS-RBD expressed in HEK cells and *E.coli*, respectively were performed as described previously ^52^. The presence of the C-terminal hexahistidine tags on the recombinant proteins was demonstrated by ELISA. For this purpose, recombinant proteins [2 µg/ml] were coated overnight in PBS onto NUNC Maxisorb 96 well plates (Thermofisher). Plates were washed 3 times with washing buffer (PBS, 0.05% Tween 20) and subsequently blocked for 3 hours with blocking buffer (PBS, 0.05% Tween 20, 2% BSA). Serial dilutions (1:1000, 1:2000 and 1:4000) of anti-His antibodies (Dianova, Hamburg, Germany) or sera obtained from rabbits immunized with either PreS-derived peptides or recombinant PreS protein were applied and incubated over night at 4°C. For the assessment of human IgG reactivity, sera from historic controls (n=10) or convalescent COVID-19 patients (n=10) were applied in a 1:50 dilution. Bound mouse, rabbit and human IgG antibodies were detected with 1:1000 diluted HRP-linked anti-mouse IgG_1_ antibodies (GE Healthcare, Marlborough, MA, USA), 1:1000 diluted HRP-linked anti-rabbit IgG antibodies (GE Healthcare, Marlborough, MA, USA) and 1: 1000 diluted HRP-conjugated anti-human IgG antibodies (BD, San Jose, CA, USA) respectively and binding was visualized with ABTS as substrate (Sigma-Aldrich, St. Louis, MO, USA). Optical densities corresponding to bound antibodies were measured at 405/492 nm with an Infinite F50 ELISA reader (Tecan, Männedorf, Swizerland). All determinations were performed in duplicates and each result is an average of duplicate determinations with <5% difference between the obtained two values.

**Immunization of rabbits and determination of RBD-specific IgG antibody responses**

HEK cell-expressed folded RBD and preS-RBD were adsorbed onto aluminum hydroxide (SERVA Electrophoresis, Heidelberg, Germany) resulting in two dose formulations containing, 20 µg or 40 µg protein, equimolar regarding RBD, per 0.31 mg aluminum hydroxide in a volume of 0.5 ml 10 mM NaH_2_PO_4_, 0.9 % NaCl, pH 7.2. Three rabbits per protein dose were immunized subcutaneously 3 times in a three-weekly interval (Charles River, Chatillon sur Chalaronnne, France). Serum samples from rabbits were obtained before the first immunization (D0, pre-immune sera) and on days 21, 35, 42 and 64 after the first immunization. Sera were stored at -20°C until use. The measurements of rabbit IgG antibody responses to folded HEK cell-expressed RBD (Wuhan) and to RBD variants delta and omicron (Sino Biological Inc., Beijing, P.R.China) (Table S1, Figure S1) were performed as previously described ^24^.

**Immunization of a COVID-19 naïve subject with *E. coli* and HEK cell-expressed PreS-RBD**

A healthy, non-allergic male subject without any detectable IgE sensitization when tested by ImmunoCAP ISAC technology to more than 170 allergen molecules ^53^ (RV, Austrian, age 58 years, profession medical doctor, corresponding author of this paper) started immunizing himself with PreS-RBD on October 9, 2020 when no approved COVID-19 vaccine was available in Austria. The self-experiment was carried out voluntarily and in accordance with the declaration of Helsinki with the desire to induce SARS-CoV-2-specific immunity. For the first three immunizations unfolded *E. coli*-expressed PreS-RBD was adsorbed onto aluminum hydroxide (SERVA Electrophoresis, Heidelberg, Germany) resulting in a formulation containing 80 µg protein per 0.62 mg aluminum hydroxide in a volume of 0.5 ml 50 mM NaH_2_PO_4_, 10mM Tris, 20 mM, HEPES, 0.9 % NaCl, pH 4.5. For the following series of immunizations folded HEK cell-expressed PreS-RBD was adsorbed onto aluminum hydroxide resulting in a formulation containing 120 µg protein per 0.31 mg aluminum hydroxide in a volume of 0.5 ml 10 mM NaH_2_PO_4_, 0.9 % NaCl, pH 7.2. Figure 3 provides a detailed overview of the time points/dates of immunization and sample collection performed in the volunteer. The volunteer performed 3 subcutaneous immunizations with unfolded *E. coli*-expressed PreS-RBD on day 0, day 29 and day 64. Approximately six months (i.e., 196 days) after the third injection with unfolded *E. coli*-expressed PreS-RBD, the subject performed 3 subcutaneous immunizations with HEK cell-expressed folded PreS-RBD in intervals of three weeks.

Blood sampling was performed by venipuncture at 20 time points (i.e., visits 1-20) displayed in Figure 3. At two time points (visit 15 and visit 18, Figure 3) mucosal fluids were collected. Nasal fluids were collected by nasosorption (FX-I, Hunt Developments Limited, UK) and performed as described by Leaker et al. ^54^ and eluted in 300µl PBST. Tears were collected by Schirmer test (sterile Schirmer filter paper strips 5x35 mm, Haag-Streit-UK, Essex, UK) ^55^. For this purpose, the filter paper was placed for 5 minutes in the lower eyelid until the paper was completely soaked and subsequently eluted in 300 µl PBST. Mucosal fluids were stored at -80°C until analysis.

**Detection of SARS-CoV-2-specific antibody responses in serum samples from the volunteer and human subjects**

IgG, IgA, IgM and IgG_1-4_ antibody levels specific for RBD, PreS, and IgG to RBD variants were measured by ELISA as described ^24^. In brief, proteins were coated (2µg/ml) over night. After blocking, serum samples were applied in different dilutions (i.e., 1:50 to 1:800 as indicated in the experiments). Bound antibodies were detected as described ^24^ and are given as optical density (OD) values corresponding to amounts of bound antibodies. All determinations were performed in duplicates and each result is an average of duplicate determinations with <5% difference between the two measured results.

Reactivity of human IgG, IgA, IgM antibodies specific for micro-arrayed SARS-CoV-2 proteins and 25-30mer peptides spanning the S protein was determined in serum samples (1:50 diluted) and mucosal fluids (1:5 diluted) obtained at different indicated time points was performed as described. Specific antibody levels are indicated in ISAC standardized units (ISU) ^24^. Likewise, IgG antibody levels specific for eight overlapping peptides spanning the complete PreS sequence and peptides comprising the consensus PreS amino acid 13–51 sequences of HBV genotypes A–H ^30^ were determined by microarray technology ^24, 56^.

Quantitative determination of S1-specific IgG in serum samples was performed using an Anti-SARS-CoV-2-QuantiVac-ELISA kit (Euroimmun, Lübeck, Germany) according to manufacturer’s instructions and results are calculated as binding antibody units (BAU)/ml.

**Virus neutralization tests and molecular inhibition assay**

Two types of SARS-CoV-2 virus neutralization tests were performed. The first SARS-CoV-2 neutralization test utilized a SARS-CoV-2 isolate obtained in Austria at the beginning of the pandemic and is based on the measurement of the cytopathic effect of the virus on cultured Vero E6 cells (ATCC CRL-1586). In this assay the assessment of virus neutralization titers of plasma samples with 50–100 TCID_50_ SARS-CoV-2 was done as described ^24, 57^.

The second SARS-CoV-2 virus neutralization assay is based the determination of a 50% reduction in the anti-SARS-CoV-2 NP staining. This assay was performed with the human SARS-CoV-2 isolate BetaCoV/Munich/BavPat1/2020, which was kindly provided by Christian Drosten, Charité, Berlin ^58^ and distributed by the European Virology Archive (Ref-SKU: 026V-03883). The virus was passaged once through the human lung adenocarcinoma cell line Calu-3 (ATCC HTB-55^TM^) to obtain a high titer virus stock. Neutralization assays were performed according to Amanat el al. ^59^. Briefly, one day prior the assay, aliquots of 10.000 Vero cells (ATCC CCL-81^TM^) were seeded into each well of a 96-well plate containing Dulbecco´s Modified Eagle´s medium; DMEM, Gibco/Thermo Fisher, high glucose, with GlutaMAX and sodium pyruvate, supplemented with 10% fetal calf serum; FCS, Biowest, Nuaillé, France; 1% MEM Non-Essential Amino Acids Solution, Gibco/Thermo Fisher; 100 U/mL penicillin and 100 μg/mL streptomycin, Gibco/Thermo Fisher. On the next day, sera were heat-inactivated (56°C for 30 min) and serially diluted in a DMEM medium with reduced serum (2% FCS) and incubated in duplicates with the SARS-CoV-2 virus (80 μL, equalling 800 half-maximal tissue culture infectious dose (TCID50) per well; final volume 160 μl per well) for 1 hour at 37 °C in a biosafety level 3 facility of the Medical University of Vienna. After 1 hour, 120 μL of the mixture was used to infect the Vero cell monolayers, achieving infection of 600 TCID50/well. After 48 hours incubation, infected cells were fixed with 10% formaldehyde in PBS, followed by the 5% formaldehyde post-fixation. Subsequently, the cells were washed with PBS, permeabilized with 0.1% Triton X-100 in PBS, blocked with blocking buffer (10% FCS in PBS+0.05% Tween-20), and stained with rabbit anti-SARS-CoV-2 NP antibodies (40143-R019, SinoBiological, Beijing, China) diluted 1:15000 in blocking buffer), followed by the HRP-labelled goat anti-rabbit antibodies (170-6515, Bio-Rad, diluted 1:10000 in blocking buffer). The In-Cell ELISA was then developed using the DY999 substrate solution (R&D Systems, Minneapolis, MN) and measured at 450 nm (and 630 nm for the background) using a Mithras multimode plate reader (Berthold Technologies, Bad Wildbad, Germany). Titers were calculated in GraphPad Prism 9 by generating a 4-parameter logistical fit of the percent neutralization at each serial serum dilution. The 50% virus neutralization titer (VNT50) was reported as the interpolated reciprocal of the dilution yielding a 50% reduction in the anti-SARS-CoV-2 NP staining.

The molecular interaction assay to detect inhibition of RBD binding to ACE2 by patients´ plasma samples was performed as described ^23,24^ with either 100 ng or 50 ng of HEK cell-expressed folded RBD. It reports the percentage of inhibition of either 100 ng or 50 ng RBD binding to ACE2 by blocking antibodies, respectively.

**Assessment of cellular reactivity**

Peripheral blood mononuclear cells (PBMC) were isolated from heparin anti-coagulated blood which was diluted 1:2 with IMDM (Hyclone, Cytiva, Pasching, Austria) medium containing 20 U/ml heparin, 10 % FCS (Gibco, Thermo Fisher Scientific, Carlsbad, CA), 15 µg/ml Gentamicin and 0.5 µg/ml Amphotericin. The so prepared blood samples were gently overlaid onto Ficoll-Hypaque gradients in 50 ml tubes followed by centrifugation at 500 g for 15 minutes. The PBMC-rich interphase was collected, washed twice with fresh medium and re-suspended at a concentration of 1x10^7^ cells/ml in IMDM+20% FBS +10% DMSO for freezing. Portions of 1x10^7^ cells were gently transferred into a Cryotube (Nunc, Thermo Fisher Scientific, Carlsbad, CA) and frozen in an isopropanol filled Mr. Frosty (Nalgene, Sigma Aldrich, St. Louis, MO) at -80°C. After at least 48h at -80°C, samples were transferred in liquid Nitrogen until analyses.

Immunophenotyping was carried out on freshly obtained cells according to standard procedures as shown previously ^60^. In particular, B cell subpopulations were gated and analyzed according to the EUROclass trial ^61^. Intracellular staining was performed according to standard procedures ^~~60~~^ ^62^ and as described previously ^60^.

CD4+ and CD8+ SARS-CoV-2-specific T cell responses were determined in frozen PBMCs obtained from the volunteer at visits 9, 11, 14, 17 and 19 (Figure 3) and from vaccinated controls approximately 4 weeks after the last vaccination (Table S2). Thawing was performed by gently swirling for 2 minutes in a 37°C water bath and dropwise adding of pre-warmed RPMI 1640 medium (Lonza, Basel, Switzerland) supplemented with 10% FBS. Cells were washed two times with 15 ml pre-warmed medium via centrifugation for 10 minutes at 300xg. Subsequently aliquots of 3x10^6^ cells/ml were carboxyfluorescein succinimidyl ester (CFSE) stained ^63^. Fluorescent dye-labelled cells were cultured and stimulated with either HEK cell expressed RBD (60 nM) or equimolar quantities of a mix of nine overlapping peptides spanning RBD ^24^. Additionally cells of the subject were stimulated with PreS and a PreS-peptide mix as described ^29^. Unstimulated cells or Dynabeads® Human T-Activator CD3/CD28 (1 μg/well (Invitrogen, Carlsbad, CA)) stimulated cells served as negative and positive controls, respectively. Cells were cultured for seven days at 37°C and 5% CO_2_ and subsequently stained with a PerCP/Cy5.5-labelled monoclonal anti-human [CD3 antibody](https://www.sciencedirect.com/topics/medicine-and-dentistry/cd3-antibody) (BioLegend, San Diego, CA), Brilliant Violet 421™ labeled monoclonal anti-human [CD4 antibody](https://www.sciencedirect.com/topics/medicine-and-dentistry/cd4-antibody) (BioLegend) and an APC-labelled anti-human CD8 antibody (BioLegend) and Fixable Viability Dye eFluor® 780 (BioLegend). Isotype controls: PerCP/Cy5.5 mouse [IgG2a](https://www.sciencedirect.com/topics/medicine-and-dentistry/immunoglobulin-g2a) (BioLegend), Brilliant Violet 421™ mouse IgG_1_ (BioLegend), APC mouse (BioLegend) were performed as described ^29^. Flow Cytometry was performed on a BD FACS Canto II (Becton, Dickinson and Company, Franklin Lakes, NJ). Analysis was performed via [FlowJo](https://www.sciencedirect.com/topics/biochemistry-genetics-and-molecular-biology/sequest) Software, Version 10. Results represent means of triplicate cultures and median percentages of proliferated CD3^+^CD4^+^ and CD3^+^CD8^+^ above medium background are shown for the different antigens and subjects.

**Statistical analysis**

No statistical methods were used to predetermine sample sizes. All statistical analyses were performed using GraphPad Prism Version 5.00 (La Jolla, CA, USA). Correlations of immunoglobulin reactivity and virus neutralization titers were assessed by Spearman´s rank correlation coefficient. p values of <0.05 were considered as significant.

**References Methods**

52. Resch Y, Weghofer M, Seiberler S, et al. Molecular characterization of Der p 10: a diagnostic marker for broad sensitization in house dust mite allergy. *Clin Exp Allergy.* 2011;41(10):1468-1477. doi:10.1111/j.1365-2222.2011.03798.x

53. Lupinek C, Wollmann E, Baar A, et al. Advances in allergen-microarray technology for diagnosis and monitoring of allergy: the MeDALL allergen-chip. *Methods.* 2014;66(1):106-119. doi:10.1016/j.ymeth.2013.10.008

54. Leaker BR, Malkov VA, Mogg R, et al. The nasal mucosal late allergic reaction to grass pollen involves type 2 inflammation (IL-5 and IL-13), the inflammasome (IL-1β), and complement. *Mucosal Immunol.* 2017;10(2):408-420. doi:10.1038/mi.2016.74

55. Aghayan-Ugurluoglu R, Ball T, Vrtala S, Schweiger C, Kraft D, Valenta R. Dissociation of allergen-specific IgE and IgA responses in sera and tears of pollen-allergic patients: a study performed with purified recombinant pollen allergens. *J Allergy Clin Immunol.* 2000;105(4):803-813. doi:10.1067/mai.2000.104782

56. Niespodziana K, Stenberg-Hammar K, Megremis S, et al. PreDicta chip-based high resolution diagnosis of rhinovirus-induced wheeze. *Nat Commun.* 2018;9(1):2382. Published 2018 Jun 18. doi:10.1038/s41467-018-04591-0

57. Koblischke M, Traugott MT, Medits I, et al. Dynamics of CD4 T Cell and Antibody Responses in COVID-19 Patients With Different Disease Severity*. Front Med (Lausanne).* 2020;7:592629. Published 2020 Nov 11. doi:10.3389/fmed.2020.592629

58. Rothe C, Schunk M, Sothmann P, et al. Transmission of 2019-nCoV Infection from an Asymptomatic Contact in Germany. *N Engl J Med.* 2020;382(10):970-971. doi:10.1056/NEJMc2001468

59. Amanat F, White KM, Miorin L, et al. An In Vitro Microneutralization Assay for SARS-CoV-2 Serology and Drug Screening. *Curr Protoc Microbiol.* 2020;58(1):e108. doi:10.1002/cpmc.108

60. Kratzer B, Trapin D, Ettel P, et al. Immunological imprint of COVID-19 on human peripheral blood leukocyte populations. *Allergy.* 2021;76(3):751-765. doi:10.1111/all.14647

61. Wehr C, Kivioja T, Schmitt C, et al. The EUROclass trial: defining subgroups in common variable immunodeficiency. *Blood*. 2008;111(1):77-85. doi:10.1182/blood-2007-06-091744

62. Cossarizza A, Chang HD, Radbruch A, et al. Guidelines for the use of flow cytometry and cell sorting in immunological studies (second edition). *Eur J Immunol.* 2019;49(10):1457-1973. doi:10.1002/eji.201970107

63. Quah BJ, Warren HS, Parish CR. Monitoring lymphocyte proliferation in vitro and in vivo with the intracellular fluorescent dye carboxyfluorescein diacetate succinimidyl ester. *Nat Protoc.* 2007;2(9):2049-2056. doi:10.1038/nprot.2007.296

**Supplemental Tables and Figure legends**

**Table S1.** Characteristics of subjects vaccinated with licensed COVID-19 vaccines.

| **ID** | **Gender** | **Age** | **Vaccine**  **first dose** | **Side effects** | **Vaccine**  **second dose** | **Side effects** | **Blood sampling**  **days after full immunzation^1^** |
| --- | --- | --- | --- | --- | --- | --- | --- |
| A077 | f | 27 | Janssen COVID-19 Vaccine | fever | n.a. | n.a. | **28** |
| C019 | m | 28 | Janssen COVID-19 Vaccine | fever | n.a. | n.a. | **28** |
| A287 | m | 34 | Vaxzevria | fever, fatigue, arthralgia | Vaxzevria | fatigue, conjunctivitis | **27** |
| A292 | f | 60 | Vaxzevria | fever, fatigue | Vaxzevria | none | **29** |
| A286 | m | 30 | Comirnaty | urticaria | Comirnaty | none | **27** |
| A288 | m | 55 | Comirnaty | none | Comirnaty | none | **27** |
| A291 | f | 33 | Comirnaty | fatigue | Comirnaty | fatigue | **31** |
| A290 | m | 39 | Comirnaty | none | Comirnaty | none | **27** |
| A289 | m | 31 | Vaxzevria | fever, fatigue, arthralgia | Comirnaty | fatigue | **26** |

^1^ Full immunization is defined as application of two doses of Comirnaty (Pfizer/BionTech) and Vaxzevria (AstraZeneca) or one dose of Janssen COVID-19 vaccine (Johnson & Johnson) according to initial European Medical Agency, EMA, application of the respective vaccines.

**Table S2.** Distribution of lymphocyte subsets in peripheral blood upon PreS-RBD immunization of subject.

| Visit | Immunization with | CD45RO+CCR7+ in CD3+CD4+ | CD45RO+CCR7+ in CD3+CD8+ | CD21- B lymphocytes | Plasmablasts IgM- CD38++ | CD10- CD21-  B cells |
| --- | --- | --- | --- | --- | --- | --- |
| V1 | PreS-RBD [*E.coli*] | 18.48 | 7.76 | 3.68 | 0.37 | 0.87 |
| V2 |  | 25.49 | 16.48 | 4.21 | 0.81 | 0.94 |
| V3 |  | 16.27 | 7.73 | 2.9 | 0.36 | 0.85 |
| V4 | PreS-RBD [*E.coli*] | 23.18 | 8.85 | 4.01 | 0.51 | 1.16 |
| V5 |  | 15.38 | 2.92 | 3.59 | 1.02 | 1.44 |
| V6 | PreS-RBD [*E.coli*] | 16.28 | 4.77 | 3.37 | 1.22 | 1.12 |
| V7 |  | 21.96 | 9.47 | n.d. | n.d. | n.d. |
| V8 |  | 20.3 | 5.53 | 3.22 | 0.58 | 1.41 |
| V9 | PreS-RBD [*HEK*] | n.d. | n.d. | n.d. | n.d. | n.d. |
| V11 |  | 41.29 | 24.28 | 15.72 | 13.09 | 10.67 |
| V12 |  | 14.73 | 5.36 | 3.88 | 0.71 | 1.87 |
| V13 | PreS-RBD [*HEK*] | 14.33 | 2.12 | 6.66 | 1.05 | 2.34 |
| V14 |  | 15.91 | 2.42 | 4.78 | 1.92 | 2.1 |
| V15 |  | 18.41 | 3.37 | 4 | 0.44 | 1.8 |
| V17 | PreS-RBD [*HEK*] | 18.42 | 3.95 | 2.99 | 0.8 | 1.24 |
| V18 |  | 15.83 | 3.61 | 5.75 | 1.53 | 2.46 |
| V19 |  | 9.1 | 1.87 | 3.86 | 0.47 | 1.9 |
| V20 |  | n.d. | n.d. | n.d. | n.d. | n.d. |

n.d., not done

**Figure S1.**  Visualization of amino acid exchanges in the RBD of models made for the S protein structures of the SARS-CoV-2 variants. Shown are top views of the S protein (surface representation) with amino acid exchanges in color (bottom) of **(A)** alpha (B.1.1.7), **(B)** beta (B.1.351), **(C)** gamma (P.1), **(D)** delta (B.1.617.2) and (**E**) omicron (B.1.1.529).

**Figure S2.** Sequence alignment of SARS-CoV-2 RBD (Wuhan, Genbank accession Nr.: QHD43416.1) with RBD variants. The amino acid sequence of RBD from the SARS-CoV-2 Wuhan strain (top) has been aligned with the amino acid sequences from RBD variants (alpha, beta, gamma, delta and omicron, see footnote). Different amino acids are indicated, identical amino acids are indicated by dots and positions in the S protein are given on the margins. The sequences were colored to illustrate conserved features in addition to amino acid identity (light red = acidic hydrophilic, yellow= neutral, basic hydrophilic= light green, hydrophobic = light blue).

**Figure S3.** IgG responses to RBD (Wuhan) and RBD variants (delta, omicron) (inset) in **(A)** 1:50 diluted serum samples from the subject immunized with folded PreS-RBD at indicated time points or in **(B)** 1:1000 diluted sera from six rabbits (numbered 7-12) obtained three weeks after immunization with two doses (equimolar to 20 or 40 µg of RBD) folded PreS-RBD. OD values (y-axes) are averages of duplicate determinations with <5% deviation and correspond to bound antibodies.

**Figure S4.** Time courses of RBD and PreS-specific antibody responses in the immunized subject. IgG-, IgM-, IgA-levels specific for folded RBD (left) and PreS (right) in different dilutions of serum (insets) obtained at different time points after immunization (indicated with black star) (x-axes) with folded HEK-cell expressed RBD-PreS measured by ELISA. OD (405/492 nm) values represent the averages of duplicate determinations with <5% deviation (y-axes) and correspond to levels of bound antibodies.

**Figure S5.** PreS-specific antibodies in the immunized subject. Shown are IgG levels (y-axes: ISU) to **(A)** eight overlapping peptides (preS P1-P8) spanning the whole preS sequence of the HBV genotype A.2 and to **(B)** peptides of amino acid 13–51 in HBV genotypes A–H consensus sequences as determined by microarray technology at three indicated time points (V1, V9 and V20). ISU values (y-axes, means of triplicates) correspond to specific IgG antibody levels.

**Figure S6.** S- and RBD-specific IgG levels of subjects vaccinated with licensed SARS-CoV-2 vaccines. Shown are IgG levels to folded S and folded RBD measured by ELISA at different serum dilutions (inset) 4 weeks after full immunization, according to initial European Medical Agency approval, of a random sample of healthy subjects (x-axes, Table S1) with SARS-CoV-2 vaccines or buffer alone. OD (405/492nm) values (y-axes) are averages of duplicate determinations with <5% deviation and correspond to bound antibodies.

**Figure S7.** Time courses of **(A)** IgG, **(B)** IgA and **(C)** IgM antibody levels specific for the indicated SARS-CoV-2 antigens and peptides spanning the RBD (insets) (y-axes: mean ISU, ISAC-standardized units, of triplicates) as determined by microarray technology in serum samples (1:50) obtained at different time points (x-axes) after immunization of the subject with unfolded *E. coli*-expressed (white stars) or folded HEK cell-expressed (black stars) PreS-RBD.

**Figure S8.** Microarray-based analysis of **(A)** IgG-, **(B)** IgA- and **(C)** IgM antibody levels specific for SARS-CoV-2 antigens and peptides spanning the RBD (insets) in mucosal fluid samples obtained at visit 15 and visit 18 (nasal secretion: left; tears: right). ISU (ISAC-standardized units) values (y-axes) correspond to antibody levels.

**Figure S9.** Induction of plasmablasts approximately 7 days after initial vaccination and after booster injections with folded PreS-RBD in the subject. **(A)** Gating strategy for the identification of B cells in whole blood. B cells were identified according to their FSC-A and SSC-H profile, further gated as singlets based on their distinct fluorescence signal obtained by FSC-W versus FSC-H. Finally they were identified by gating on CD45+ positive leukocytes and CD19+ lymphocytes. **(B)** Percentages of IgM-CD38++ plasmablasts (++ superhigh, i.e., CD38high) within the CD19+ B cell compartment at the indicated time points (top). White stars indicate immunizations with unfolded *E. coli* and black stars with folded HEK cell-expressed PreS-RBD. Abbreviations used in the figure: SSC-H, side scatter height; FSC-H, forward scatter area; FSC, forward scatter height; FSC-W, forward scatter width (time of flight); eF506, eFluor 506 nM; PE-eF610, tandem dye phycoerythrin plus eFluor 610 nM; eF405, eFluor 405 nM; eF610, eFluor 610 nM; APC-eF780, allophycocyanine eFluor 780 nM.

**Figure S10.** Specific CD4^+^ and CD8^+^ T cell responses in the immunized subject and in subjects immunized with licensed vaccines. **(A)** RBD and RBD-peptide,- PreS- and PreS-peptide-specific CD4^+^ (left) and CD8^+^ (right) T cell responses (y-axes: percentages of proliferated T cells after subtraction of medium control) in the subject vaccinated with PreS-RBD at different time points (x-axes). **(B)** RBD and RBD-peptide-specific CD4^+^ (left) and CD8^+^ (right) T cell responses (y-axes: percentages of proliferated T cells after subtraction of medium control) in subjects vaccinated with licensed vaccines (x-axes).

**Figure S11**. Correlation of **(A)** standardized antibody binding units (y-axis: BAU/ml) with RBD-specific IgG levels measured by ELISA (x-axis: OD levels) and with **(B)** percentages inhibition of RBD-ACE2 binding (x-axis) using 100 ng (left) or 50 ng (right) of RBD (x-axis). **(C)** Correlation of VNT50 titers (y-axes) with percentages inhibition of RBD-ACE2 binding using 100 ng (left) or 50 ng (right) of RBD (x-axes). **(D)** Correlation of percentage of inhibition of RBD-ACE2 binding (y-axis) using 100 ng (left) or 50 ng (right) of RBD with VNTs (x-axis). Correlations were calculated for samples from Table 1 for which paired results were available for the parameters which were compared. r and p values are indicated.
